# Arsenic, cadmium, lead, antimony bioaccessibility and relative bioavailability in legacy gold mining waste

**DOI:** 10.1016/j.jhazmat.2024.133948

**Published:** 2024-03-05

**Authors:** Farzana Kastury, Julie Besedin, Aaron R. Betts, Richmond Asamoah, Carina Herde, Pacian Netherway, Jennifer Tully, Kirk G. Scheckel, Albert L. Juhasz

**Affiliations:** a Future Industries Institute, STEM, University of South Australia, SA, Australia; b School of Science, STEM, RMIT University, Victoria, Australia; c United States Environmental Protection Agency, Center for Environmental Solutions and Emergency Response, Land Remediation and Technology Division, Cincinnati, OH, USA; d South Australian Health and Medical Research Institute, Adelaide 5086, Australia; e EPA Science, Environment Protection Authority Victoria, Centre for Applied Sciences, Ernest Jones Drive, Macleod, Melbourne, Victoria 3085, Australia; f United States Environmental Protection Agency, Center for Environmental Solutions and Emergency Response, Water Infrastructure Division, Cincinnati, OH, USA

**Keywords:** Bioaccessibility, Bioavailability, Relative bioavailability, Speciation, Gold mining

## Abstract

Bioaccessibility and relative bioavailability of As, Cd, Pb and Sb was investigated in 30 legacy gold mining wastes (calcine sands, grey battery sands, tailings) from Victorian goldfields (Australia). Pseudo-total As concentration in 29 samples was 1.45–148-fold higher than the residential soil guidance value (100 mg/kg) while Cd and Pb concentrations in calcine sands were up to 2.4-fold and 30.1-fold higher than the corresponding guidance value (Cd: 20 mg/kg and Pb: 300 mg/kg). Five calcine sands exhibited elevated Sb (31.9–5983 mg/kg), although an Australian soil guidance value is currently unavailable. Arsenic bioaccessibility (n = 30) and relative bioavailability (RBA; n = 8) ranged from 6.10–77.6% and 10.3–52.9% respectively. Samples containing > 50% arsenopyrite/scorodite showed low As bioaccessibility (<20.0%) and RBA (<15.0%). Co-contaminant RBA was assessed in 4 calcine sands; Pb RBA ranged from 73.7–119% with high Pb RBA associated with organic and mineral sorbed Pb and, lower Pb RBA observed in samples containing plumbojarosite. In contrast, Cd RBA ranged from 55.0–67.0%, while Sb RBA was < 5%. This study highlights the importance of using multiple lines of evidence during exposure assessment and provides valuable baseline data for co-contaminants associated with legacy gold mining activities.

## Graphical Abstract.

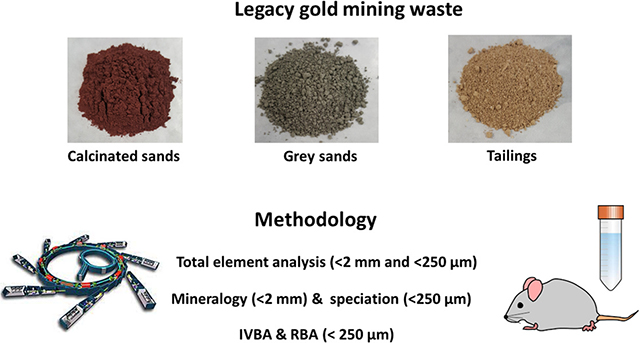

## 1. Introduction

Gold mining and arsenic (As) contamination are reliably entwined because of the role As geochemistry plays in gold accumulation in iron-sulfide hosted gold systems. Formation of arsenopyrite (FeAsS) from pyrite (FeS_2_) via multistage hydrothermal events may enhance the adsorption of gold-complexes from fluid onto pyrite surfaces; As and gold are then transported together and recrystallize in metamorphic sediments [1,2]. Because of this close association, gold mining activities have resulted in widespread As contamination in many legacy goldfields around the world. Several epidemiological studies have linked gold mining related As exposure to negative health outcomes, e.g., high cancer risk and adverse birth outcomes were reported in Banmauk, Myanmar and Northern Tanzania [3,4] and high non-carcinogenic risk was identified in Eastern Amazon, Brazil [5]. As a result, investigations into human health risk from gold mining impacted waste material exposure remains an important global environmental issue.

Victorian goldfields (Australia) hosted one of the world’s largest orogenic gold deposits, yielding at least 2,500 tons of gold between 1851–1914, which is ~30% of all Australian gold and ~2% of the world’s gold supply [6,7]. Nearly 40% of all gold from Victoria was alluvial, depositing in sand/gravel of streambeds due to the weathering of nearby orogenic deposits [7]. However, the majority of gold was recovered by crushing and processing subsurface quartz deposits [6]. Refuse materials from mining processes were generally discarded in waterways as sediment or ‘sludge’, causing substantial detriment to townships, industries and the environment downstream of mining activities [8]. Waste materials associated with gold mining can be broadly categorized as tailings (generic waste materials from ore crushing), grey slimes [fine grey colored particles, produced either from stamp batteries or as sludge/clay settling at the bottom of separation tanks (hereafter termed “Grey battery sands”)] and calcinated materials [resulting from high temperature roasting (~500 °C) of Fe-rich flotation concentrate (hereafter termed “Calcine sands”)] [9,10].

When gold was hosted within arsenopyrite (FeAsS), roasting converted As into arsenic trioxide (As_2_O_3_) vapors, which precipitated as As_2_O_3_-rich dust [11]. Although the majority of the As_2_O_3_ may be released into the atmosphere via the roasting process, calcine sands may contain elevated As (>10,000 mg/kg) residues [9,11–15]. In Victoria, the mining board introduced sludge control regulations from 1858, while Victoria’s Board of Health advocated for As_2_O_3_ emission reduction strategies from 1872 (e.g., furnace redesign and recapturing/repurposing As_2_O_3_ as a pesticide) [8,10]. However, variable efficacies of these environmental regulations during six decades of gold mining deposited large volumes of mine wastes into the environment. The seminal early work of Hinwood et al., [16] reported that As concentrations in Victorian goldfields ranged between 1.70 – 16,800 mg/kg, which continues to pose environmental and human health risk [17–21].

Although As is the major contaminant in mine impacted waste in the Victorian goldfields, other co-contaminants [e.g., cadmium (Cd), lead (Pb) and antimony (Sb)] may also be present at elevated concentrations. For example, Ollson et al., [9] found up to 12.8 mg/kg Cd and 1,810 mg/kg Pb in the < 250 μm particle size fraction (calcine sands and tailings), Martin et al., [12] reported 1,150 mg/kg Sb in the < 2 mm particle fraction (calcine sands), while Kastury et al., [14] identified 1, 302 mg/kg Pb in the < 10 μm particle size fraction (calcine sands). Similarly elevated co-contaminants in legacy gold mining impacted matrices have also been reported globally, e.g., Sb in Yellowknife, Canada [11] and east Otago, New Zealand [22], Cd and Pb in Delita, Cuba [23] and Castromil, Portugal [24]. While these co-contaminants may not be present in every mine waste type, their identification in the above-mentioned studies suggest that a comprehensive assessment of co-contaminants is needed to understand the overall exposure risk for inhabitants of legacy gold mining regions.

Limited studies have investigated human health exposure to As and co-contaminants in gold-mining impacted wastes via *in vitro* bioaccessibility assay (IVBA: elemental dissolution using simulated gastrointestinal solutions) and/or relative bioavailability assays (RBA: elemental absorption into systemic circulation relative to absorption from a water-soluble form) [25,26]. Variability in IVBA and RBA of potentially toxic co-contaminants among different waste materials and factors contributing towards this variation remains an important knowledge gap for contaminated soil exposure assessment. As such, this study aimed to 1) assess elemental concentrations in legacy mining waste from Victorian goldfields to identify elements of concern 2) investigate variability in IVBA and RBA among mine wastes type and among co-contaminants, and 3) determine elemental speciation which may contribute to IVBA and RBA variability.

## 2. Materials and methods

### 2.1. Sample collection, processing, and categorization

Gold mining waste (0–20 cm, ~1–2 kg) was collected from 30 different sites across Victorian Goldfields, dried (40 °C) and sieved to < 2 mm. Categorization of samples in three mine waste types was performed according to Ollson et al., [9] using a soil color chart [27]. Samples exhibiting bright purple color were categorized as “Calcine sands” (n = 7: C1-C7). The remaining samples were further divided into ‘Grey battery sands’ (grey colored samples with fine particles, n = 11: G1-G11) and ‘Tailings’ (brown/yellow-colored samples with visibly coarser particles, n = 12: T1-T12). Particle sizing was undertaken by hand sieving to determine the mass of particles in the < 250 and < 53 μm particle size fractions. For IVBA and RBA assessment, the < 250 μm particle size fraction was utilized as this is the upper size fraction that adheres to hands / fingers that is available for incidental ingestion.

### 2.2. Sample characterization

Sample pH was measured in MilliQ water [sample (g): water (mL) = 1:5, n = 3]. The < 2 mm and < 250 μm particle fractions (0.5 g; n = 2) were pre-digested in 5 mL aqua-regia [36% hydrochloric acid (HCl): 70% nitric acid (HNO_3_) = 3:1] overnight. Digestion was conducted using a Mars6 microwave (CEM) using USEPA protocol 3051 A [28], followed by syringe filtration (0.45 μm, cellulose acetate) and storage at 4 °C. A standard reference material (SRM) from the National Institute of Standards and Technology (NIST 2710a) was used during digestion to determine digestion accuracy (see information in Section 2.5. quality assurance and quality control). Pseudo-total elemental concentration was determined using Inductively Coupled Plasma (ICP) Optical Emission Spectrometry (OES) using Method 6010D [29]. Elements analyzed included Aluminium (Al), As, Barium (Ba), Calcium (Ca), Cd, Cobalt (Co), Chromium (Cr), Iron (Fe), Magnesium (Mg), Manganese (Mn), Nickel (Ni), Pb, Sb, Strontium (Sr), Titanium (Ti), Vanadium (V) and Zinc (Zn). Total Sulfur (S) was determined using CNS (Leco Trumac) (0.2 g, n = 1).

Mineralogical composition of the samples (<2 mm) was assessed to provide an indication of the major mineral phases in a sample. Semi-quantitative X-ray Diffraction (XRD) analysis was undertaken using a PAnalytical Empyrean diffractometer equipped with theta (θ) – 2 theta (θ) goniometer using Cu K-α radiation of wavelength 1.54 Å and a detector. Scans were conducted from 5–120° 2θ at 0.006 2-θ intervals, a count time of 5 s/point and X-ray conditions of 40 kV and 40 mA. Mineral phase identification was conducted using the X′Pert HighScore Plus software package linked with the ICDD PDF-4 + 2022 powder diffraction file. Quantification of mineral phases was conducted using the SIROQUANT V4 package which employs the ‘Rietveld’ refinement method.

X-ray Absorption Spectroscopy (XAS) was performed on the < 250 μm particle fraction using As K-edge (11,867 eV), Pb L_III_-edge (13,035 eV) and Sb K-edge (30,491 eV) at the Materials Research Collaborative Access Team [30], Advanced Photon Source (Argonne National Laboratory). More information is given in Supplementary Information (SI). For further speciation analysis, data was collected with a JEOL JEM 6490LV scanning electron microscope (SEM) with backscatter detection (BSD). Samples were measured using accelerating voltage of 20 kV, spot size 60, high vacuum, 10 mm working distance. The energy dispersive X-ray spectrometry (EDS) system was an Oxford X-max 50 mm^2^ silicon drift detector. Images and spectra were analyzed using Aztec software.

### 2.3. In vitro bioaccessibility assessment (IVBA)

Arsenic, Cd, Pb and Sb IVBA was assessed using the < 250 μm particle fraction (n = 2) according to USEPA Method 1340 (gastric phase extraction at pH 1.5) [31]. A reference sample, SoFc-1 (n = 10), and a blank (n = 10) was used every 20 samples (see information in Section 2.5. quality assurance and quality control). At the end of the assay, dissolved elements were separated from residual solids by syringe filtration (0.45 μm, cellulose acetate) and stored at 4 °C, until analyzed by ICP-Mass Spectrometry (MS) using USEPA method 6020 A [32]. Elemental IVBA (%) was calculated using Eq. (1).


(1) $$IVBA(\%)=\frac{In\;vitro\;elemental\;concentration}{Pseudo\;-\;total\;elemental\;concentration}\times 100$$

Where:

*In vitro* elemental concentration = As/Cd/Pb/Sb (mg/kg) extracted in simulated gastric solution using *in vitro* assay USEPA method 1340 [31].

Pseudo-total elemental concentration = As/Cd/Pb/Sb concentration (mg/kg) determined using aqua regia digestion via USEPA method 3051 A [28].

### 2.4. In vivo relative bioavailability (RBA)

Selected samples (< 250 μm particle fraction, n = 8) that contained multiple co-contaminants exceeding their respective soil guidance values (HIL A) [37] were utilized to assess As, Cd, Pb and Sb RBA. *In vivo* studies were conducted using female C57BL/6 mice (4–6-week-old) with ethical and experimental approval provided by the South Australian Health and Medical Research Institute Animal Ethics Committee (application number SAM268). Animal care was compliant with the Standard Operating Procedures of the South Australian Health and Medical Research Institute, and the Guidelines for the Care and Use of Laboratory Animals in the National Research Council (NRC, 2010). Mice were housed in metabolic cages (3 mice per cage, 3 cages per sample) for a period of 9 days and supplied with AIN93G mouse chow amended with 1% (w/w) sample (< 250 μm particle size fraction). Food consumption was monitored daily by taking the difference in food hopper mass before and after filling, with cumulative food consumption calculated at the end of the study. At the end of the nine-day exposure period, mice were maintained on unamended chow for an additional day, then humanely euthanized by cervical dislocation following administration of an isofluorene / oxygen anesthetic [33]. Urine from each metabolic cage was combined and frozen (−20 °C). To assess As and Sb RBA, urine was thawed, vortexed, and 5 mL urine was pre-digested overnight in 70% HNO_3_ (5 mL). For Cd and Pb RBA assessment, liver and kidney from the same metabolic cage were combined and pre-digested in 70% HNO_3_ (5 mL). Pre-digested urine and tissue were digested using a Mars 6 Microwave (CEM) according to [33]. Accuracy of digestion was determined using NIST SRM 2976 – mussel tissue (more information in Section 2.5. quality assurance and quality control). After syringe filtration (0.45 μm, cellulose acetate), samples were stored at 4 °C until analyzed by ICP-MS using USEPA method 6020 A [32].

Urinary excretion factors (UEF) for As or Sb were calculated using Eq. (2).


(2) $$Urinary\;excretion\;fator(UEF)=\frac{Cumulative\;urinary\;excretion}{Cumulative\;dietary\;intake}$$

As and Sb RBA was calculated using Eq. (3).


(3) $$As\;or\;SbRBA(\%)=\frac{UEF\;-\;soil}{UEF\;-\;{Na}_{3}{AsO}_{4}\;\;or\;\;K\left[Sb(OH{)}_{6}\right]}\times \frac{D\;oral\;-\;{Na}_{3}{AsO}_{4}\;or\;K\left[Sb(OH{)}_{6}\right]}{D\;oral\;-\;soil}\;\times 100$$

Where:

UEF oral-mine waste= UEF for an oral As/Sb-contaminated mine waste dose.

UEF oral- Na_3_AsO_4_ or K[Sb(OH)_6_] = UEF for an oral sodium acetate: Na_3_AsO_4_ or from Ollson et al. [34] or Potassium hexahydroxoantimonate: K[Sb(OH)_6_] (Juhasz, personal communication).

D oral- Na_3_AsO_4_ or K[Sb(OH)_6_]= dose of orally administered Na_3_AsO_4_ (mg/kg) from Ollson et al. [34] or K[Sb(OH)_6_] (mg/kg) (Juhasz, personal communication).

D oral-mine waste = dose of orally administered As/Sb contaminated mine waste (mg/kg).

Cd and Pb RBA was calculated using Eq. (4).


(4) $$Pb\;or\;Cd\;RBA(\%)=\frac{liver\;or\;kidney\;-\;soil}{Oral\;-\;Pb{\left(C_{2}H_{3}O_{2}\right)}_{2}\;or{CdCl}_{2}}\times \frac{D\;oral\;-\;Pb{\left(C_{2}H_{3}O_{2}\right)}_{2}\;or\;{CdCl}_{2}}{D\;oral\;-\;soil}\times 100$$

Where:

Liver/kidney oral-mine waste = Liver/kidney concentrations of an oral contaminated mine waste dose.

Oral- Pb(C_2_H_3_O_2_)_2_ = Liver/kidney concentrations of an oral Pb (C_2_H_3_O_2_)_2_ from Ollson et al., [34].

D oral- Pb(C_2_H_3_O_2_)_2_ = Dose of orally administered Pb(C_2_H_3_O_2_)_2_ (mg/kg) from Ollson et al. [34].

D oral-mine waste = Dose of orally administered Pb / Cd contaminated mine waste (mg/kg).

Oral- CdCl_2_ = Liver/kidney concentrations of an oral CdCl_2_ from Ollson et al. [34].

D oral- CdCl_2_ = Dose of orally administered CdCl_2_ (mg/kg) from Ollson et al. [34].

### 2.5. Statistical analysis

All statistical analysis and preparation of graphs was conducted in GraphPad Prism (10.0.2 version). The relationship between total and bioaccessible elemental concentration, as well as the relationship between As bioaccessibility and RBA was analyzed using simple linear regression to generate the line of best fit and r^2^ value was used to measure the goodness of fit.

### 2.6. Quality assurance and quality control

Digestion accuracy was confirmed using NIST SRM 2710a (certified As, Cd, Pb reference values of 1,540 mg/kg, 12.3 mg/kg, and 5,520 mg/kg respectively; Sb certified value was not available). Quantitative recovery of As, Cd and Pb during digestion from SRM 2710a was 95.7%, 96.8% and 91.4% respectively (n = 8). Reference values for As, Cd, Pb and Sb in SoFC-1, used during IVBA, were 740 mg/kg, 61.2 mg/kg, 6,398 mg/kg and 1,078 mg/kg respectively. Average IVBA for As, Cd, Pb and Sb (n = 10) were 16.9%, 5.54%, 64.5% and 11.9% respectively in SoFC-1. Urine and tissue digestion accuracies were assessed using NIST SRM 2976 (certified As, Cd and Pb reference values of 13.3 mg/kg, 0.82 mg/kg, and 1.19 mg/kg respectively; Sb certified values not available). Quantitative As recovery for urine digestion (n = 2) from SRM 2976 was 97.3% for As. Quantitative As, Cd and Pb recovery for tissue (liver and kidney) digestion (n = 5) was 110%, 113% 129% respectively. Antimony concentration from NIST 2710a and 2976 were below the level of detection (0.1 μg/L).

During analysis of digests and bioaccessibility with ICP-OES and ICP-MS, duplicates and check values were run every twenty samples and the average deviation was < 10%. Samples spiked with As, Sb and Pb were run every 20 samples and average recoveries were within 30% of spiked values.

## 3. Results and discussion

### 3.1. Physicochemical characterization of Victorian goldfield samples

Among the 30 samples collected from Victorian goldfields, seven were categorized as ‘calcine sands’ (C1-C7), 11 as ‘grey battery sands’ (G1 - G11), and the remaining 12 were grouped together as ‘tailings’ (T1 - T12). Mineralogical analysis (XRD; Table S1) of the < 2 mm particle fraction identified quartz as the most abundant constituent in samples (37.0 – 90.0%), which agrees with Martin et al., [13] who previously reported the predominance of quartz (31 – 69%) in the < 53 μm particle fraction of Victorian goldfield samples. While grey battery sands contained 2.90 – 58.7% mica (biotite + ephesite + muscovite), calcine sands contained hematite as the second major phase [25.9 – 44.8%]. The presence of hematite confirms that these purple-colored materials were produced via high temperature roasting [35].

Pseudo-total elemental concentrations are listed in Table 1 (< 2 mm) and Tables S2–S4 (< 2 mm and < 250 μm). Arsenic concentrations in the < 2 mm particle fractions ranged from 95.0 – 14,836 mg/kg (median: 2,192 mg/kg). Twenty nine of the 30 samples exceeded 100 mg/kg, which is the National Environmental Protection Measure for the Assessment of Site Contamination (NEPM-ASC) soil Health Investigation Level (HIL) A (standard residential areas with soil accessible to children with <10% fruit/vegetable intake from garden) [37]. Median As concentration (9,843 mg/kg) was the highest in calcine sands (range 3,303 – 14,836 mg/kg], followed by grey battery sands (median 2,067 mg/kg, range: 681 – 5,089 mg/kg), and tailings (median 820 mg/kg, range: 95.0 – 5,481 mg/kg). These As concentrations are similar to ranges reported by Ollson et al., [9], Martin et al., [12], and Hinwood et al., [16] (up to 47,100 mg/kg) in soils / mine wastes from the Victorian goldfields. Results also confirmed that the most elevated As concentrations were typically associated with calcine sands, which was also observed in Ollson et al., [9] and Martin et al., [12]. An epidemiological study by Hinwood et al., [19] on residents from the Victorian goldfields identified that individuals potentially exposed to soil containing > 100 mg/kg As (from residential soil exposure) were significantly associated with increased As urinary excretion (Spearman coefficient: 0.67). Hinwood et al., [19] highlighted that mean urinary As concentration doubled from 1.64 μg/L for all participants to 2.46 μg/L for participants living in proximity to soil that contained > 1,000 mg/kg As. In a follow up study by Pearce et al., [21], participants exposed to soil As concentrations between. 1.40 – 1,857 mg/kg showed a small but significant increase in past cancer risk associated with high soil As concentrations in socioeconomically disadvantaged areas. These studies suggest that calcine and grey battery sands may pose the greatest risk to human health owing to the highest As concentrations.

When surveilling for other co-contaminants of interest, calcine sands were also found to exceed the HIL A for Pb (300 mg/kg) and Cd (20 mg/kg). All seven calcine sands exceeded the Pb HIL A, ranging between 892 mg/kg (C7) - 9,023 mg/kg (C4), while three calcine sands exceeded the Cd HIL A (C4: 47.2 mg/kg Cd, C6: 20.2 mg/kg Cd and, C7: 27.6 mg/kg Cd). Although a soil guidance value has not been derived for Sb in Australia, due to structural and functional similarity between As and Sb, Sb toxicity has been compared to that of As [36]. Therefore, a guideline value of 100 mg/kg was adopted for Sb in this study, which was exceeded in four calcine sands [524 ± 20.4 (C6) - 5983 ± 20.4 (C5)]. One tailings sample exceeded the HIL A investigation level of 4, 000 mg/kg for Mn [4,730 ± 60 mg/kg (T4)]. Elevated co-contaminant concentrations in calcine sands may be an additional health concern in Victorian goldfields because Pb, Cd and Sb exposure have been associated with attention deficit/hyperactivity and autism spectrum disorders [38,39]. Also, co-exposure to Mn and Pb has been shown to exacerbate neurological impairment in children, particularly exposure during the formative years [40].

Concentrations of major cations varied widely among the < 2 mm particle fraction of the three mine waste types. Median Fe concentration was elevated in calcine sands (median: 196 g/kg, range: 17.7 – 275 g/kg), but was similar between grey battery sands (median: 28.5 g/kg, range: 22.6 – 33.9 g/kg) and tailings (median: 30.9 g/kg, range: 14.5 – 54.4 g/kg). High Fe concentrations in calcine sands was expected because Fe oxides are known to dominate roaster derived materials; however, it is noteworthy that the median Fe in calcine sands in this study was 2.7-fold higher than that the median value of 71.4 g/kg reported in Ollson et al., [9]. High variability in other major cations was also observed across the three different mine waste categories (Al: 5.76 – 41.6 g/kg, Ca: 0.19 – 46.9 g/kg and Mg: 0.49 – 41.9 g/kg). Depending on pH, As species and surface area, cations such as Al, Fe and Mn provide surfaces for As sorption and may play a significant role in lowering its dissolution and mobility, hence influence potential human health exposure [9,41]. Future studies should focus on assessing reactive Al/Fe/Mn concentrations, which may provide further insight into As and co-contaminant exposure.

### 3.2. Assessment of As exposure

#### 3.2.1. Bioaccessibility and speciation

**Fig. 1.**
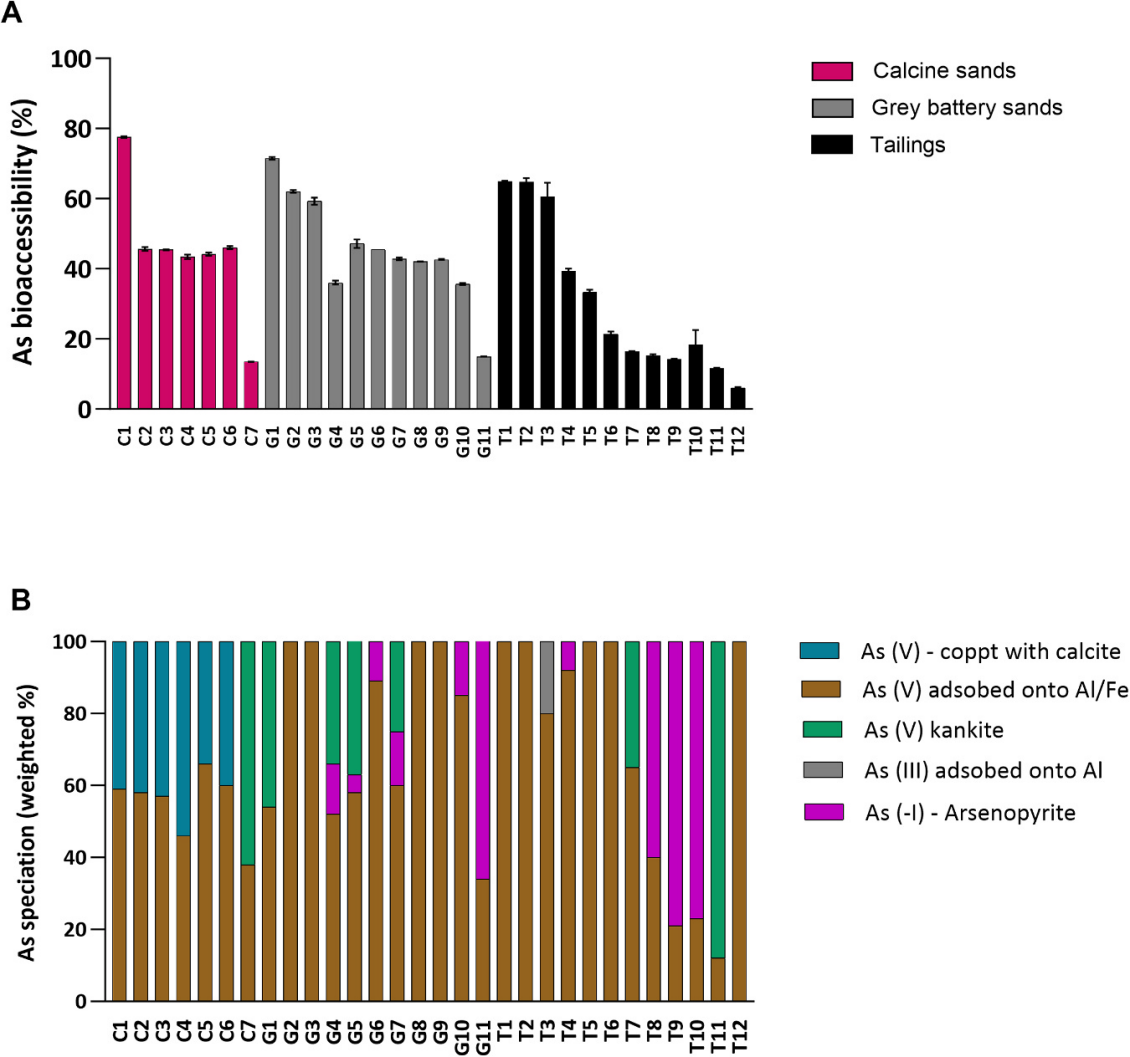
Arsenic (As) bioaccessibility (A), determined using USEPA method 1340, and As speciation (B), determined using EXAFS LCF in legacy gold-mining waste from the Victorian goldfields.

Because As soil guideline value was exceeded, further investigation into human health exposure was conducted using the incidentally ingestible < 250 μm particle fraction. Assessment of IVBA via USEPA method 1340 (gastric phase extraction at pH 1.5) was undertaken because this method was shown to be a strong predictor of As RBA due to the strengths of the *in vivo-in vitro* correlation (IVIVC, r^2^_>_0.8) reported in previous research by Diamond et al., [42], Juhasz et al., [43], Bradham et al., [44], Juhasz et al., [45]. Arsenic bioaccessibility (Fig. 1A) in the < 250 μm particle fraction showed high variability (range: 6.10 – 77.6%), with calcine and grey battery sands showing higher median bioaccessibility (45.4% and 42.8% respectively) than tailings (19.9%). High variability in As bioaccessibility is similar to previous reports of gastric phase As bioaccessibility in this region, including 5.00 – 36.0% (n = 8) in Juhasz et al., [46], 25.0 – 42.0% (n = 11) in Smith et al., [47] and 4.00 – 90.0% (n = 50) in Ollson et al., [9]. High variability was also evident in studies from other gold mining regions of the world. For example, an As bioaccessibility range of 2.86 – 73.6% was reported by Whitacre et al., [48] in gold mining impacted soils (n = 19) from California, USA using the California Bioaccessibility (CAB) method [modified from Ohio State University - *in vitro* gastrointestinal (OSU-IVG) method]. Similarly, using the gastric phase of the Solubility Bioaccessibility Research Consortium (SBRC) method, Bromstad et al., [11] observed an As bioaccessibility range of 29.0 – 40.0% in outcrop samples from Yellowknife, Canada (n = 3), while Meunier et al., [49] reported a range of 4.19 – 48.3% in soils and tailings from Nova Scotia, Canada (n = 26). However, although studies reporting As bioaccessibility based on the three mining waste categories used in this study is limited, Ollson et al., [9] reported that grey battery sands showed higher bioaccessibility (range: 70 – 90%, mean = 82%) than calcine sands (range: 25 – 84%, mean = 48%), which contrasts results from this study. The reason for this discrepancy is unclear, however, As speciation has been shown to be a major influencer of As bioaccessibility [9,25].

To gain a deeper understanding for the variability in As bioaccessibility, As XAS LCF analysis was performed on the < 250 μm particle fractions for phase identification from which we can often infer expected solubility and RBA. Arsenic speciation (weighted %) results are given in Fig. 1B, with further information regarding linear combination fitting provided in Figs. S5 and S6 and Tables S6–S8. Analysis of the 1st derivative XANES was performed as a simple, robust identification of oxidation state and relative abundance (Fig S5; Table S3). The source As species was arsenopyrite as As(-I), which during roasting and processing is oxidized to As (III) or As (V). Oxidation of As in samples showed residual As(-I) (5–79%) present in 6 of the 11 grey battery sands and 4 of the 12 tailings and none of the calcine sands. A single tailings sample, T3 contained 30% As(III) with the remaining samples contained only As(V).

Speciation analysis utilizing EXAFS LCF identified the major As species in samples as As (III or V) adsorbed onto Al/Fe oxides, scorodite (FeAsO_4_⋅2 H_2_O), arsenopyrite (FeAsS), and a hornesite-like phase (Mg_2_(AsO_4_)_3_⋅2 H_2_O) in calcine sands. High proportion of As(V) adsorbed onto Al or Fe oxides was found across most samples which was expected in oxic soils and is typically associated with moderate As bioaccessibility [9,25].

The commonly identified As species in calcine wastes from gold ore processing are arsenolite, scorodite, or As(V) incorporated hematite [36]. However, calcine sands in this study contained unique speciation likely due to the high heat transformation during the ore roasting process, which warranted additional characterization by SEM-EDS and XRD (Figs. S7 and S8) to clarify the unique phases found. Imaging and EDS mapping indicated that As was concentrated in discrete particles and only associated with Mg and minor amounts of trace elements; Fe, Pb, Sb lacked co-location with As. Analysis of XRD patterns indicated the presence of an anhydrous Mg_3_(AsO_4_)_2_ in samples C2-C6 (Fig. S8). When As XAS LCF was modelled in samples C2-C6, the results showed a mixture of hornesite, a hydrated magnesium arsenate and arsenate adsorbed to Fe oxide surfaces. Calcines from ore containing arsenopyrite typically contain oxidized residues like arsenolite (As_2_O_3_) or inclusions in maghemite or hematite or residual arsenopyrite [36,50]. However, magnesium arsenate is stable between 600–1,100 °C [51] and can form from a melt of MgCO_3_ and As_2_O_3_ which possibly occurred in the calcination process.

The highest As bioaccessibility in calcine sands was found in C1 (77.6%). C1 lacked XRD crystalline As minerals like the MgAsO_4_ found in C2-C6 (Fig. S6), despite having a similar composition and Mg content. This may be explained if the magnesium arsenate formed was poorly-crystalline, resulting in higher solubility and RBA. The C1 material also had a high proportion of < 53 μm sized particles (Fig. S2), which may also contribute to increased solubility [9,49]. In contrast, C7 contained 62% scorodite, which is a crystalline ferric arsenate (FeAsO_4_⋅2 H_2_O), generally found in sulphate rich, low pH mining sites [52–54]. Although rarely reported in alkaline soil, scorodite was also observed in four other grey sands (G1, G4, G5 and G7) and two tailings (T7 and T11), all exhibiting pH between 6.6 – 9.4. The presence of scorodite in these samples may be a result of past acidic conditions, or microclimate and water chemistry. Arsenic solubility from ferric arsenate may depend on the degree of crystallinity, with decreasing solubility with increasing crystallinity [55]. For example, the solubility product (pK_sp_) of poorly crystalline amorphous ferric arsenate is − 23.0 ± 0.3, while that of highly crystalline scorodite is − 25.8 ± 0.07 [56]. It is likely that the low As bioaccessibility in C7 (13.5%) was a result of high scorodite content in this sample. However, despite having similar proportions of Al/Fe sorbed As and scorodite, As bioaccessibility in G1 was 5.3-fold higher than C7. Low As bioaccessibility (15.0–16.4%) in G11, T8, T9 and T10 may be attributed to the presence of arsenopyrite (60–79%), which exhibits low solubility under gastric phases conditions [25].

It is unclear why As bioaccessibility varied widely (6.1–33.5%) among G8, G9, T5, T6 and T12, despite containing 100% As(V) adsorbed onto Al/Fe. This lack of explanatory value from As speciation data towards As bioaccessibility was also observed in mine waste containing a majority of adsorbed As(V) species [25]. Arsenic solubility may depend on factors other than As speciation such as Fe speciation, As: amorphous Fe molar ratio and reactive Fe concentration [9]. Additionally, physical factors could inhibit As solubility such as glass occlusions during roasting or precipitation layering. In samples containing most species as surface-bound, it is conceivable that a combination of these factors played a greater role in influencing As bioaccessibility than As speciation alone.

#### 3.2.2. Arsenic relative bioavailability (RBA) and in vivo - in vitro correlation (IVIVC)

**Fig. 2.**
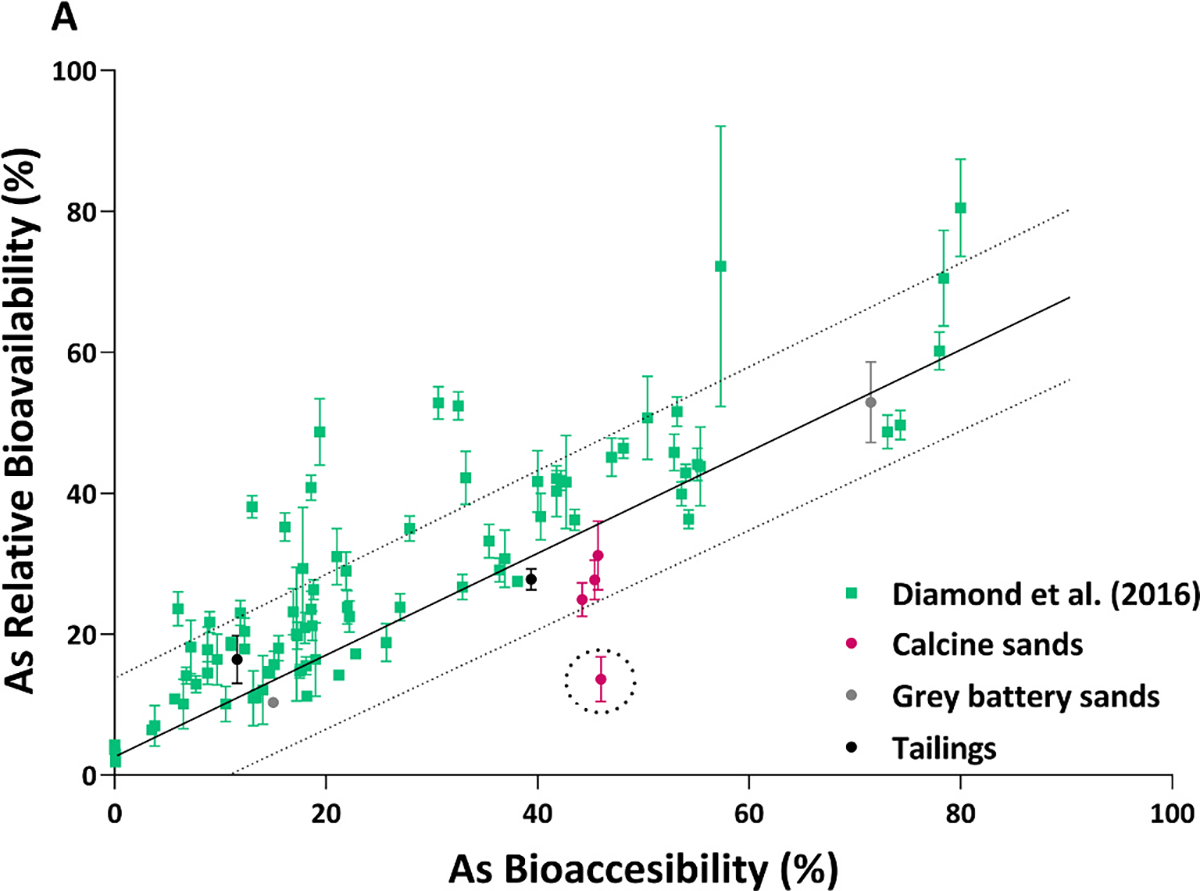
Relationship between As relative bioavailability (RBA) and bioaccessibility (IVBA) using simple linear regression to generate the line of best fit and r^2^ value was used to measure the goodness of fit. The purple, grey and black circles represent paired As RBA-IVBA data from 4 calcine sands, 2 grey sands and 2 tailings respectively (this study), while the green squares represent data from Diamond et al. (2016) that was used to derive the *in vivo - in vitro* correlation between As RBA and IVBA using USEPA method 1340. The *in-vivo-in-vitro* relationship is indicated by the solid black line (the line of best fit) and the 95^th^ percentile is indicated by the dashed lines. The datum highlighted by a circle of dashed line represents the value (C6) that falls below the 95^th^ percentile.

Although USEPA method 1340 has been validated for the prediction of As RBA [42–45], sample specific antagonistic or synergistic effects between elements during absorption in the small intestine may influence RBA outcomes [34]. Therefore, *in vivo* investigations were conducted on selected samples (n = 8; four calcine sands: C2, C3, C5, C6, two grey battery sands: G1, G11 and two tailings: T4, T11), representing a broad range of As and co-contaminant concentrations. Because the highest As concentration in gold mining waste (14,836 mg/kg) was ~2-fold higher than samples (6,899 mg/kg) used in Diamond et al., [42], the robustness of the IVIVC was re-assessed for gold mining wastes. Fig. 2 shows As RBA for the 8 samples from this study, in comparison to the RBA-IVBA outcomes from Diamond et al., [42]. Results from this study showed that As RBA was variable, ranging from 10.3–52.9%. Despite showing similar bioaccessibility and speciation, As RBA from the four calcine sands was significantly different (p = 0.0013, ANOVA). Arsenic RBA was 2-fold lower in C6 (13.6 ± 3.2%) compared to the other calcine sands (C2: 31.2 ± 4.9%, C3: 27.7 ± 2.8% and C5: 24.9 ± 2.4%). Low As RBA in C6 may be attributable to differences in Ca concentration between calcine sands (7.10 g/kg in C6 versus 2.17–5.45 g/kg in C2-C5). Conceivably, lower As RBA in C6 may result from Ca-arsenate precipitation during the *in vivo* transition from the gastric to intestinal phase [57]. A similar result was reported in Li et al., [58] where negative correlations between As RBA and Ca concentrations (r^2^_=_ 0.46) was reported for As impacted rice. As previously mentioned, high arsenopyrite content may have led to low As RBA in G11 (10.3 ± 0.7%) [59] compared to G1 (52.9 ± 5.7%), which was also evident in bioaccessibility outcomes. Lower As RBA in T11 (16.4 ± 3.4%) compared to T4 (27.8 ± 1.5%) was a result of higher scorodite content in the former, which also followed the pattern observed in bioaccessibility assays.

The study by Diamond et al., [42] detailed a model for predicting As RBA using USEPA method 1340 using the equation: As RBA (%) = 0.79 × As bioaccessibility (%) + 3.0 (r^2^_=_ 0.87; n = 83). With the exception of one calcine sand (C6), As RBA outcomes from this study were within the 95% confidence limits of the IVIVC reported in Diamond et al., [42] (Fig. 2). Although As concentrations in calcine sands were 16.7- and 30.4-fold higher than the mean (718 mg/kg) and median (399 mg/kg) As concentration used in the study of Diamond et al., [42], data were still within the 95% confidence limits. Therefore, Fig. 2 suggests that while C6 fell below the 95% confidence limits of the IVIVC, utilization of USEPA method 1340 would produce a conservative estimate of As RBA.

### 3.3. Co-contaminant exposure assessment

**Fig. 3.**
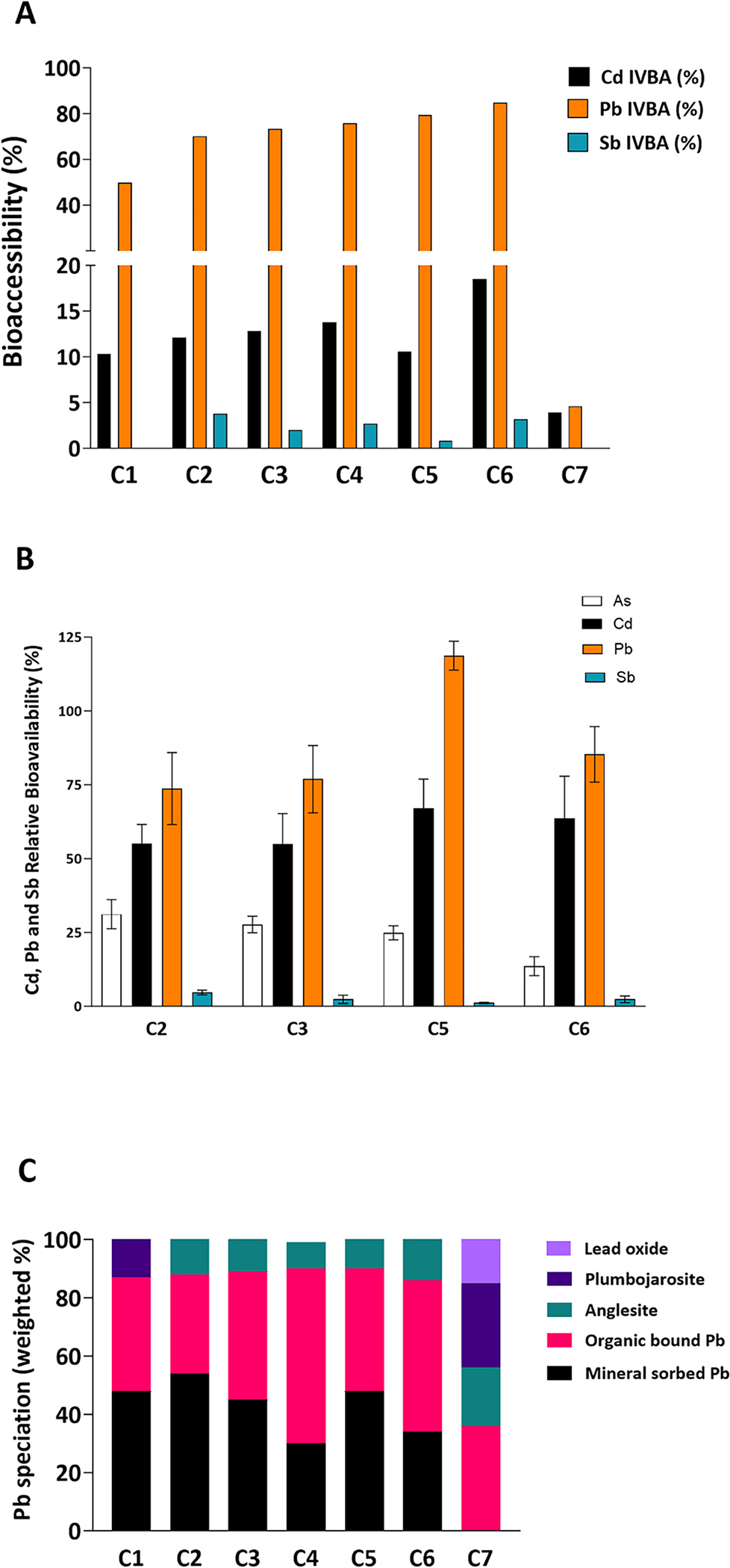
Assessment of co-contaminant bioaccessibility and speciation in calcine sands from the Victorian goldfields. Fig. 3A shows Cd, Pb and Sb bioaccessibility in all seven calcine sands, while Fig. 3B shows As, Cd, Pb and Sb RBA in four calcine sands. Fig. 3C shows Pb speciation using X-ray absorption near edge spectroscopy (XANES) in all 7 calcine sands with > 100 mg Pb/kg.

In addition to As, USEPA method 1340 has been shown to be a strong predictor of Pb RBA due to the strengths of the IVIVC [60], although limited studies have evaluated its use for predicting Cd RBA [61] and no studies have investigated its relationship to Sb RBA. Therefore, although the focus of *in vitro* and *in vivo* assessment was to determine As exposure, Pb, Cd and Sb bioaccessibility and RBA was also quantified simultaneously when concentrations were comparable to or in excess of soil guidance values. Unlike As, co-contaminants of interest (i.e., Cd, Pb and Sb) were elevated only in calcine sands, hence exposure to these elements was analyzed in calcine sands alone. Fig. 3A and B depicts Cd, Pb and Sb bioaccessibility (%) and RBA (%) respectively, while the relationships between their total and bioaccessible concentrations and linear regression analysis are provided in Fig. S10. However, it is noteworthy that due to the small number of samples with bioaccessibility outcomes, this relationship may need to be investigated in future studies with larger sample sizes to verify reproducibility. Additionally, to illustrate the influence of speciation on bioaccessibility outcomes, weighted % of Pb species are also shown in Fig. 3C. Speciation assessment was not undertaken for Cd due to its low total concentration. Further information about the linear combination fitting for Pb and Sb is given in Figs. S11–S12 and Tables S9–S10.

#### 3.3.1. Co-contaminant bioaccessibility and speciation

Among the three co-contaminants, Pb bioaccessibility (4.60 – 84.9%) was the highest in calcine sands (Fig. 3A) and was strongly correlated to total Pb concentration in the < 250 μm particle fraction (Fig. S9; r^2^: 0.98, p < 0.0001). All calcine sands contained two major phases, organic bound Pb (34–52%), and mineral sorbed Pb (30–54%), with anglesite (PbSO_4_), lead oxide (PbO) and plumbojarosite $\left[{Pb}_{0.5}{Fe}_{3}^{3+}{\left({SO}_{4}\right)}_{2}(OH{)}_{6}\right]$ as minor phases (Fig. 3C). Organic matter bound Pb may exhibit moderate Pb solubility because organic molecules act as a complexing agent for Pb [62], while mineral sorbed Pb tends to solubilize readily when exposed to acidic pH (1.5) of the gastric phase of USEPA method 1340 [63,64]. Additionally, anglesite is highly soluble in both acidic and neutral pH (2− 7) [65]. Therefore, high Pb bioaccessibility in C2-C6 (i.e., > 70%), where organic bound, mineral sorbed Pb and anglesites were the only phases, can be attributed to the moderate - high solubility of these phases. Although Pb exposure reports from gold mining waste are rare, high Pb bioaccessibility (58.9%) in the < 10 μm particle size fraction of a calcine sand containing 48% mineral sorbed Pb and 52% organic bound Pb was reported in Kastury et al., [14]. In contrast, lower Pb bioaccessibility in C1 (49.8%) and C7 (4.64%) can be attributed to the presence of plumbojarosite (14% and 29% respectively), a Pb-Fe mineral which exhibits low solubility under gastric solutions [66–69]. It is noteworthy that C7 showed exceptionally low As and Pb bioaccessibility compared to other calcine sands, which suggests that formation of plumbojarosite and scorodite for Pb and As respectively can greatly reduce bioaccessibility for both elements.

Fig. 3A shows that Cd bioaccessibility was low, ranging from 3.90 – 18.5% (median 12.1%) with a significant and strong relationship to total Cd concentration in the < 250 μm particle fraction (Fig. S9; r^2^: 0.67, p < 0.05). Although no other study using gold-mining waste has reported Cd bioaccessibility, the results of this study can be compared to findings in Li et al., [70], where using the same assay conditions, high Cd bioaccessibility (35.0 – 107%) was observed in 12 soils from diverse contamination sources (e.g., mining/smelting, farming and residential activities). However, soils used in Li et al., [70] contained higher total Cd concentrations (3.00 – 269 mg/kg) and lower total Al, Fe and Mn concentrations in the < 2 mm particle fractions (0.74 – 5.88 g/kg, 1.76 – 45.5 g/kg, and 2.58 – 8,463 mg/kg respectively). This suggestis that the higher Al+Fe+Mn:Cd ratio in this study most likely contributed to stronger sorption of Cd to minerals, resulting in low bioaccessibility.

Similarly, low Sb bioaccessibility was observed in the five Sb containing calcine sands (Fig. 3A, range: 0.80–3.80%, median: 2.70%). Similar to Cd, this is the first study reporting Sb bioaccessibility from gold mining waste. Among the limited studies reporting Sb bioaccessibility, Denys et al., [71] utilized the Unified BARGE bioaccessibility (UBM) method to assess mining/smelting (n = 15) impacted soils containing 18 – 60,000 mg Sb/kg. In these soils, Sb bioaccessibility was < 20% which was attributed to Sb binding to sulfides, oxy-hydroxides, and refractory soil constituents. However, Sb speciation analysis in the four calcine sands with *>* 100 mg/kg Sb (C2, C3, C5 and C6) revealed that 100% of the Sb was present as Sb(V) in these four samples, which excludes low Sb bioaccessibility being the result of Sb sulfides, which is predominantly a form of Sb(III). Using extended X-ray absorption fine structure (EXAFS), Mitsunobu et al., [72] and Ilgen and Trainor [73] showed that Sb(V) forms strong inner sphere complexes with clay and Al/Fe oxides [(e.g., bidentate mononuclear and binuclear complexes shown in Vithanage et al., [74]]. Therefore, low Sb bioaccessibility may have resulted from inner sphere complexes of this element with Al/Fe/clay. While bioaccessibility studies are limited for Sb impacted matrices, Cappuyns et al., [75] reported low Sb solubility following Sb leaching behavior assessment from mine waste, slag, and soil samples near slag heaps (47 – 15,699 mg/kg Sb, n = 8). Cappuyns et al., [75] leached samples in a range of pH representing acidic, neutral and alkaline solutions such as deionized water, solutions of calcium chloride (CaCl_2_) and disodium phosphate (Na_2_HPO_4_). Antimony solubility was found to be < 1% in Cappuyns et al., [75], which was attributed to strong sorption to humic acids/Fe oxides and the age of the mine waste type contributing to the increased Sb sorption to the matrix. It is noteworthy that among the three co-contaminants, the relationship between total and bioaccessible Sb was not significant (p > 0.05, Fig. S9), although future studies with higher number of Sb containing samples are needed to verify the robustness of this relationship, as well as to elucidate factors contributing to Sb bioaccessibility.

#### 3.3.2. Co-contaminant relative bioavailability

Similar to bioaccessibility outcomes, co-contaminant RBA (Fig. 3B) was the highest for Pb (73.7 – 119%), followed by Cd (55.0 – 67.0%) and Sb (1.20 – 4.70%). Despite having similar total Pb concentration, bioaccessibility and speciation, Pb RBA in C5 was 1.4- to 1.6-fold higher than the other calcine samples. Competition with cations during absorption, presumably via the divalent cation transporter, was reported to lower Pb absorption [76,77]. However, the combined molar ratio of Al+Ca+Cd+Fe+Mg+Mn: Pb was C2 > C5 > C3 > C6, which does not agree with the observed Pb RBA pattern, prompting investigation into the effect of individual elements on Pb RBA. Among these cations, Ca:Pb molar ratio was between 12.6 to 14.9 for C2, C3 and C6, while for C5 it was 6.54. Therefore, it is possible that increased competition with Ca via the divalent metal transporter contributed to lower Pb RBA in C2, C3 and C6 compared to C5 [76], although this ratio is most likely to be influenced by the reactive Ca concentrations, instead of total Ca concentrations. Similarly, it is also conceivable that high Ca or Pb concentrations in calcine sands may similarly lower Cd RBA. However, Cd RBA values were 3.4–6.3-fold higher than bioaccessibility values derived using USEPA method 1340, which is typically the opposite to the results observed by previous Cd RBA investigations [61]. The reason for this difference is unclear although future studies assessing Cd speciation may elucidate factors influencing IVIVC. Similar to low Sb bioaccessibility outcomes, Sb RBA was also low in all four samples (1.2–4.7%), which is in agreement with the low Sb RBA (<11%) reported previously in Denys et al., [71] following the assessment of mining/smelting impacted soils. The low Sb RBA may be attributed to the aforementioned strong inner sphere complexes of Sb(V) with Al/Fe/Clay.

## 4. Conclusions

The fundamental processes controlling exposure of toxic elements from legacy goldfields bear significant implications for global human health risk management. Among the elements of concern, As was found to be elevated in most mining waste included in this study from the Victorian goldfields. In a series of previous studies, elevated As concentrations in this region was shown to increase urinary As output and incorporation into nails, leading to a small increase in cancer risk at Victorian goldfields [17–19]. Similar reports around the world corroborates the suggestion that significant human health risk is associated with As from gold mining waste [4,5,78]. Additionally, although it is well established that co-contaminant concentrations (e.g., Cd, Pb and Sb) may be elevated in soils as a result of gold mining activities, this is the first study to assess co-contamination exposure using *in vitro* and *in vivo* assessment methods in gold mining waste. Elevated concentrations of these co-contaminants in Victorian goldfields raise considerable concern because Pb, Cd and Sb exposure have been associated with attention deficit/hyperactivity and autism spectrum disorders [38,39], while co-exposure to Mn and Pb has been shown to exacerbate neurological impairment in children, particularly during the formative years [40]. Therefore, development of strategies for the effective remediation of mine waste (e.g., contaminant immobilization) need to focus not only on As, but also extend to co-contaminant stabilization, which often shows antagonistic effects with As immobilization outcomes [64].

Although both bioaccessibility and RBA assays were utilized in this study as surrogate measures for assessing exposure to multiple inorganic environmental pollutants, IVIVC for co-contaminants in gold mining wastes (e.g., Cd and Sb) needs further investigation to understand the strength of the predictive relationship using USEPA method 1340. Furthermore, major cations (e.g., Ca) were observed to influence absorption of these contaminants and this information may be utilized during the development of dietary strategies for exposure reduction. Overall, this study highlighted the strength of using multiple lines of evidence approach during exposure assessment and provided an important baseline for As and co-contaminant exposure associated with legacy gold mining activities in the Victorian goldfields.

## Supplementary Material

Supplementary Material

## Figures and Tables

**Table 1:** Soil pH and selected trace and major element concentrations in the < 2 mm particle fraction of mining waste. All elements were analysed using ICP-OES using duplicates, except S, which was assessed using CNS without a replicate. ‘HIL A’ refers to the Health Based Investigation Level A according to the National Environment Protection Measure for the Assessment of Site Contamination (NEPM 2013). NA = not applicable. ‘LOD’ represents the limit of detection during analysis using ICP-OES and values below this limit is indicated as ‘< LOD’.

Sample name		Soil pH	Trace elements					Major elements				
			As (mg/kg)	Cd (mg/kg)	Pb (mg/kg)	Mn (mg/kg)	Sb (mg/kg)	Al (g/kg)	Ca (g/kg)	Fe (g/kg)	Mg (g/kg)	S (mg/kg)

**HIL A**			100	20	300	4000	*NA*	*NA*				
**Instrumental LOD**			0.1	0.1	0.1	0.1	0.0001	0.0001	0.0001	0.0001	0.0001	
**Calcine sands**	**C1**	8.1	8249 ± 30.8	17.7 ± 0.21	1418 ± 8.31	628 ± 0.78	< LOD	17.3 ± 0.51	5.28 ± 0.01	194 ± 1.74	5.87 ± 6.37	700
	**C2**	8.0	14,836 ± 156	18.5 ± 0.07	1742 ± 9.67	875 ± 6.32	776 ± 21.8	22.2 ± 0.13	5.25 ± 0.03	204 ± 1.44	9.92 ± 0.05	1100
	**C3**	7.7	12,868 ± 112	18.9 ± 0.04	2047 ± 5.65	884 ± 1.09	1284 ± 11.8	20.8 ± 0.14	5.45 ± 0.06	196 ± 0.22	8.75 ± 0.06	1500
	**C4**	7.2	9843 ± 211	47.2 ± 0.78	9023 ± 177	915 ± 38.1	31.9 ± 4.52	12.4 ± 1.60	2.38 ± 0.07	245 ± 2.34	5.22 ± 0.97	1100
	**C5**	7.1	8666 ± 0.35	18.1 ± 0.04	1714 ± 8.71	984 ± 22.7	5983 ± 20.4	20.3 ± 0.97	2.17 ± 0.02	177 ± 0.70	6.08 ± 0.54	700
	**C6**	8.2	10,088 ± 427	20.2 ± 0.37	2922 ± 52.3	1199 ± 77.9	524 ± 20.4	19.8 ± 1.51	7.10 ± 0.34	179 ± 3.53	6.62 ± 0.16	800
	**C7**	8.3	3303 ± 140	27.6 ± 0.14	892 ± 23.0	57 ± 2.85	< LOD	5.76 ± 154	0.95 ± 0.20	276 ± 0.70	0.49 ± 0.013	700
**Grey sands**	**G1**	7.7	2542 ± 6.57	< LOD	125 ± 11.2	373 ± 8.39	< LOD	27.4 ± 0.31	5.24 ± 0.01	32.3 ± 0.27	11.6 ± 0.31	1000
	**G2**	7.6	2791 ± 12.0	2.23 ± 0.02	45.2 ± 0.04	373 ± 9.57	< LOD	25.7 ± 0.41	8.94 ± 0.25	33.6 ± 0.02	10.9 ± 0.23	1800
	**G3**	7.8	1653 ± 11.9	< LOD	104 ± 4.36	267 ± 5.37	< LOD	21.4 ± 1.62	6.16 ± 0.07	27.2 ± 0.22	14.3 ± 0.17	5200
	**G4**	7.8	681 ± 34.5	< LOD	49.7 ± 4.36	307 ± 5.33	< LOD	9.79 ± 0.52	8.64 ± 0.03	28.5 ± 0.99	7.45 ± 0.20	1800
	**G5**	7.9	2316 ± 21.7	2.09 ± 0.02	38.9 ± 1.77	366 ± 2.42	< LOD	26.8 ± 5.59	6.08 ± 0.22	31.3 ± 0.78	8.74 ± 0.14	700
	**G6**	8.5	1038 ± 2.65	< LOD	70.6 ± 1.36	336 ± 5.75	< LOD	11.3 ± 0.26	4.21 ± 0.05	23.8 ± 0.64	5.29 ± 0.03	700
	**G7**	9.0	2924 ± 57.6	1.89 ± 0.03	33.2 ± 1.36	339 ± 0.11	5.39 ± 0.08	17.0 ± 6.74	7.17 ± 0.06	26.5 ± 0.13	7.25 ± 0.02	800
	**G8**	7.9	1816 ± 33.4	2.41 ± 0.08	41.2 ± 0.78	396 ± 25.8	< LOD	22.6 ± 0.58	6.71 ± 0.02	33.0 ± 0.34	10.7 ± 0.25	900
	**G9**	7.7	1420 ± 1.71	1.66 ± 0.09	28.8 ± 1.54	331 ± 8.15	2.64 ± 2.64	19.4 ± 0.29	6.15 ± 0.37	23.6 ± 0.40	7.44 ± 0.23	900
	**G10**	7.5	2067 ± 34.1	1.83 ± 0.04	45.4 ± 0.56	333 ± 13.5	< LOD	18.6 ± 2.41	6.45 ± 0.04	24.7 ± 0.31	6.83 ± 0.17	900
	**G11**	7.8	5089 ± 652	2.19 ± 0.03	44.2 ± 0.04	570 ± 7.76	< LOD	11.8 ± 1.53	12.4 ± 0.04	33.9 ± 0.47	4.91 ± 0.013	700
**Tailings**	**T1**	9.4	843 ± 16.5	< LOD	< LOD	382 ± 1.95	< LOD	27.3 ± 0.89	46.9 ± 6.95	16.8 ± 0.50	41.9 ± 6.25	1600
	**T2**	9.4	800 ± 1.88	< LOD	9.4 ± 0.53	203 ± 0.88	< LOD	35.9 ± 1.94	21.4 ± 0.36	18.4 ± 0.32	43.3 ± 01	1500
	**T3**	8.7	469 ± 6.67	2.04 ± 0.25	< LOD	341 ± 0.29	< LOD	26.4 ± 0.74	51.5 ± 0.76	14.5 ± 0.85	55.7 ± 1.59	1000
	**T4**	8.1	2530 ± 8.45	2.44 ± 0.10	145 ± 1.29	4730 ± 60	7.31 ± 0.31	19.5 ± 1.07	157 ± 6.21	29.9 ± 0.72	22.1 ± 0.08	5400
	**T5**	7.9	593 ± 6.50	< LOD	13.2 ± 0.05	228 ± 5.95	< LOD	41.6 ± 1.11	11.3 ± 1.06	25.7 ± 0.25	18.9 ± 1.48	900
	**T6**	8.7	95 ± 2.86	< LOD	18.7 ± 1.54	110 ± 1.07	< LOD	38.7 ± 1.35	2.33 ± 0.07	34.8 ± 4.01	5.95 ± 0.13	1100
	**T7**	8.3	840 ± 27.5	2.91 ± 0.39	78.5 ± 0.82	630 ± 321	< LOD	11.0 ± 1.70	5.16 ± 0.07	41.2 ± 2.35	1.16 ± 0.06	1200
	**T8**	7.2	1566 ± 37.2	4.28 ± 0.30	32.6 ± 2.04	380 ± 5.24	< LOD	41.3 ± 1.53	2.70 ± 0.09	68.9 ± 3.59	2.30 ± 0.04	700
	**T9**	7.8	5481 ± 74.3	2.54 ± 0.06	55.5 ± 0.54	601 ± 8.59	< LOD	10.9 ± 45.6	13.8 ± 0.05	38.9 ± 1.36	6.06 ± 0.02	2700
	**T10**	7.9	4791 ± 16.6	2.49 ± 0.03	47.5 ± 1.51	569 ± 11.1	< LOD	12.0 ± 2.43	14.0 ± 0.22	39.5 ± 3.07	5.43 ± 0.05	5000
	**T11**	6.6	538 ± 1.34	2.03 ± 0.02	174 ± 5.52	157 ± 1.03	34.7 ± 5.25	19.3 ± ,2.17	0.71 ± 0.56	31.8 ± 0.14	2.97 ± 0.16	4800
	**T12**	7.0	145 ± 21.0	< LOD	84.5 ± 20.8	66 ± 9.64	< LOD	15.7 ± 6.98	0.19 ± 10.3	17.7 ± 0.59	1.02 ± 0.35	500

## Data Availability

Data will be made available on request.

## HIGHLIGHTS

- Legacy gold mining waste showed high As and co-contaminants (Cd, Pb, Sb).
- Highest As (~1.5%), Pb (~0.9%) and Sb (~0.6%) concentrations were in calcine sands.
- As RBA was 10.3–52.9%; lowest RBA occurred with the highest Ca in calcine sands.
- High Pb and Cd RBA was observed (Pb: 80.1–113%, Cd: 55.0–67.0%) in calcine sands.
- RBA for Sb in calcine sands was low (1.2–4.7%).

## Environmental implications

Soil impacted from legacy gold mining activities may contain elevated concentrations of toxic elements including As, Cd, Pb and Sb. Exposure to these contaminants is essentially controlled at the mineral-solution interface in the gastrointestinal tract. This interaction is largely influenced by elemental speciation but may also be affected by particle size and type/concentration of cations in soil. Therefore, to understand human health exposure, comprehensive characterization of soils was performed followed by exposure assessment using *in vitro* and *in vivo* methodologies. This knowledge has direct applications in developing effective strategies to reduce human health exposure, as well as for environmental remediation.
