# Supplementary material for: Arsenic, cadmium, lead, antimony bioaccessibility and relative bioavailability in legacy gold mining waste
Source: J Hazard Mater. Author manuscript; Available in PMC 2024 May 16. (PMC11097331; doi:10.1016/j.jhazmat.2024.133948)
Supplement: Supplementary Material [file NIHMS1989503-supplement-Supplementary_Material.docx]

**Arsenic, cadmium, lead, antimony bioaccessibility and relative bioavailability in legacy gold mining waste**

***Supplementary Information***

Farzana Kastury^a*^, Julie Besedin^ab^, Aaron R. Betts^c^, Richmond Asamoah^a^, Carina Herde^d^, Pacian Netherway^e^, Jennifer Tully^f^, Kirk G. Scheckel^c^, Albert L. Juhasz^a^

^a^Future Industries Institute, STEM, University of South Australia, SA, Australia; ^b^School of Science, STEM, RMIT University, Victoria, Australia; ^c^United States Environmental Protection Agency, Center for Environmental Solutions and Emergency Response, Land Remediation and Technology Division, Cincinnati, Ohio, USA; ^d^South Australian Health and Medical Research Institute, Adelaide 5086, Australia; ^e^EPA Science, Environment Protection Authority Victoria, Centre for Applied Sciences, Ernest Jones Drive, Macleod, Melbourne, Victoria, 3085, Australia^f^United States Environmental Protection Agency, Center for Environmental Solutions and Emergency Response, Water Infrastructure Division, Cincinnati, Ohio, USA

^*^Corresponding author

Farzana Kastury

Future Industries Institute,

University of South Australia,

Building X, Mawson Lakes Campus, Adelaide, SA, 5095, Australia

**Phone:** +61 4 33 100 212

**Email:** farzana.kastury@unisa.edu.au

**ORCID ID:** [0000-0001-5593-0414](https://www.scopus.com/redirect.uri?url=https://orcid.org/0000-0001-5593-0414&authorId=56784375300&origin=AuthorProfile&orcId=0000-0001-5593-0414&category=orcidLink)

**Table S1:** Mineralogy of the < 2 mm mine waste particle fraction using X-ray Diffraction. * = Albite + Anthorthite. ** = Biotite + Ephesite + muscovite, *** = Ankerite + Malachite + Chalcopyrite + Dolomite. Fe-oxides are hematite except the samples indicated with “#”, which is magnetite.

|  |  | **Quartz** | **Feldspar*** | **Pumpellyite** | **Chloritoid** | **Almandine** | **Chlorite** | **Mica**** | **Kaolin** | **Carbonates***** | **Halite** | **Sulphur** | **Jarosite** | **Butlerite** | **Fe-oxides** |
| --- | --- | --- | --- | --- | --- | --- | --- | --- | --- | --- | --- | --- | --- | --- | --- |
| **Calcine sands** | **C1** | 61.9 |  |  |  |  |  |  |  |  |  |  |  |  | 38.1 |
|  | **C2** | 64.5 |  |  |  |  |  | 2.8 |  |  |  |  |  |  | 32.6 |
|  | **C3** | 66.2 |  |  |  |  |  | 2.6 |  |  |  |  |  |  | 31.3 |
|  | **C4** | 62.1 | 12 |  |  |  |  |  |  |  |  |  |  |  | 25.9 |
|  | **C5** | 60.7 |  |  |  |  |  |  |  |  |  |  |  |  | 39.3 |
|  | **C6** | 70.8 |  |  |  |  |  |  |  |  |  |  |  |  | 29.2 |
|  | **C7** | 51.1 |  |  |  |  |  | 4.1 |  |  |  |  |  |  | 44.8 |
| **Grey sands** | **G1** | 45.9 | 8.9 |  |  |  | 11 | 34.2 |  |  |  |  |  |  |  |
|  | **G2** | 49.3 |  |  |  |  | 10.2 | 40.6 |  |  |  |  |  |  |  |
|  | **G3** | 37.0 | 4 |  |  |  |  | 55.8 | 3.3 |  |  |  |  |  |  |
|  | **G4** | 67.1 | 4 |  |  |  | 6 | 23 |  |  |  |  |  |  |  |
|  | **G5** | 37.3 |  |  |  |  | 4 | 58.7 |  |  |  |  |  |  |  |
|  | **G6** | 79.1 |  |  |  |  | 1.4 | 19.5 |  |  |  |  |  |  |  |
|  | **G7** | 82.1 |  |  |  |  |  | 16.2 | 0.1 |  |  |  |  | 1.6 |  |
|  | **G8** | 54.1 |  |  |  |  | 9.3 | 34 |  |  |  |  |  | 2.5 |  |
|  | **G9** | 84.4 |  |  |  |  |  | 22.3 | 1.5 |  |  |  |  | 1.8 |  |
|  | **G10** | 75.5 |  |  |  |  | 1.4 | 14.4 |  | 8.7 |  |  |  |  |  |
|  | **G11** | 71.9 | 16.2 |  |  |  |  | 2.9 | 9 |  |  |  |  |  |  |
| **Tailings** | **T1** | 48.5 |  | 18 | 8.2 | 5.5 |  |  |  | 19.8 |  |  |  |  |  |
|  | **T2** | 94.5 |  |  |  | 5.7 |  |  |  |  |  |  |  |  |  |
|  | **T3** | 38.5 |  |  | 10.8 |  |  |  |  | 28.7 | 19.8 |  | 2.2 |  |  |
|  | **T4** | 49.6 |  |  |  |  |  |  |  | 34.7 |  | 11.1 |  |  | 4.6^#^ |
|  | **T5** | 86.2 |  |  |  | 6.3 |  |  |  | 7.6 |  |  |  |  |  |
|  | **T6** | 90.3 |  |  |  | 6.6 |  |  |  |  | 3.2 |  |  |  |  |
|  | **T7** | 75.7 |  |  |  |  | 1 | 23.3 |  |  |  |  |  |  |  |
|  | **T8** | 52.4 | 37.9 |  |  |  |  | 7.2 | 2.6 |  |  |  |  |  |  |
|  | **T9** | 48.5 | 31.8 |  |  |  |  | 18 | 1.6 |  |  |  |  |  |  |
|  | **T10** | 49.6 | 34.9 |  |  |  | 3.9 | 11.6 |  |  |  |  |  |  |  |
|  | **T11** | 75.1 |  |  | 6.9 | 4.5 |  | 3.7 | 7.7 |  |  |  |  | 2.2 |  |
|  | **T12** | 83.8 |  |  |  | 3 |  | 2.8 | 8.1 |  |  |  |  | 2.3 |  |

**Table S2:** Average ± Standard Error of the Mean (SEM) pseudo-total elemental concentrations (n = 2) in the < 2 mm, < 250 µm, < 53 µm and < 20 µm particle size fractions of calcine sands from Victorian goldfields (Australia). Values below the limit of detection is indicated as “<LOD “.

| **Elements** | **Particle size** | **C1** | **C2** | **C3** | **C4** | **C5** | **C6** | **C7** |
| --- | --- | --- | --- | --- | --- | --- | --- | --- |
| **Al (mg/kg)** | <2 mm | 17264 ± 512 | 22207 ± 128 | 20764 ± 140 | 12395 ± 1570 | 20253 ± 973 | 19771 ± 1513 | 5760 ± 154 |
|  | <250 µm | 18891 ± 156 | 19830 ± 1298 | 19233 ± 132 | 15790 ± 306 | 20936 ± 1922 | 19641 ± 318 | 6082 ± 64.0 |
| **As (mg/kg)** | <2 mm | 8249 ± 30.8 | 14836 ± 156 | 12868 ± 112 | 9843 ± 211 | 8666 ± 0 | 10088 ± 427 | 3303 ± 140 |
|  | <250 µm | 8322 ± 16.5 | 14984 ± 21.7 | 12881 ± 229 | 11865 ± 50.7 | 8782 ± 113 | 11320 ± 25.2 | 3647 ± 22.3 |
| **Ba (mg/kg)** | <2 mm | 254 ± 2.76 | 280 ± 2.22 | 266 ± 1.39 | 148 ± 3.82 | 232 ± 5.84 | 230 ± 3.33 | 33 ± 0.64 |
|  | <250 µm | 268 ± 0.08 | 265 ± 11.4 | 246 ± 1.43 | 193 ± 0.75 | 240 ± 11.2 | 255 ± 8.35 | 37 ± 0.27 |
| **Ca (mg/kg)** | <2 mm | 5283 ± 11.4 | 5247 ± 27.6 | 5451 ± 56.9 | 2375 ± 74.7 | 2168 ± 17.2 | 7100 ± 336 | 946 ± 193 |
|  | <250 µm | 5132 ± 10.1 | 5143 ± 29.1 | 5433 ± 31.2 | 2508 ± 40.6 | 2173 ± 13.1 | 6971 ± 84.7 | 1005 ± 46.1 |
| **Cd (mg/kg)** | <2 mm | 17.7 ± 0.21 | 18.5 ± 0.07 | 18.9 ± 0.04 | 47.2 ± 0.77 | 18.1 ± 0.04 | 20.2 ± 0.37 | 27.6 ± 0.14 |
|  | <250 µm | 17.5 ± 0.09 | 18.8 ± 0.05 | 18.9 ± 0.17 | 51.5 ± 0.07 | 18.0 ± 0.18 | 23.7 ± 0.04 | 28.9 ± 0.13 |
| **Co (mg/kg)** | <2 mm | 147 ± 0.56 | 165 ± 2.68 | 155 ± 0.01 | 118 ± 4.69 | 122 ± 0.43 | 130 ± 2.83 | 108 ± 1.19 |
|  | <250 µm | 145 ± 0.05 | 167 ± 0.27 | 153 ± 3.51 | 127 ± 3.78 | 123 ± 1.98 | 147 ± 1.17 | 118 ± 0.61 |
| **Cr (mg/kg)** | <2 mm | 26.6 ± 0.42 | 28.8 ± 0.07 | 29.0 ± 0.04 | 19.5 ± 2.58 | 31.4 ± 1.60 | 25.7 ± 2.00 | < LOD |
|  | <250 µm | 28.9 ± 0.15 | 26.4 ± 1.29 | 27.0 ± 0.04 | 25.7 ± 0.32 | 32.4 ± 2.63 | 26.1 ± 0.18 | < LOD |
| **Fe (mg/kg)** | <2 mm | 194150 ± 1737 | 204014 ± 1441 | 196764 ± 224 | 245082 ± 2334 | 177418 ± 70 | 178666 ± 3528 | 275848 ± 969 |
|  | <250 µm | 193517 ± 1896 | 206102 ± 576 | 194613 ± 1493 | 247324 ± 1738 | 178306 ± 945 | 192529 ± 926 | 283076 ± 329 |
| **Mg (mg/kg)** | <2 mm | 5872 ± 6.37 | 9920 ± 53.1 | 8752 ± 57.4 | 5217 ± 82.8 | 6084 ± 42.4 | 6615 ± 156 | 489 ± 12.6 |
|  | <250 µm | 6028 ± 18.6 | 9801 ± 99.4 | 8767 ± 0.47 | 6326 ± 34.6 | 6128 ± 11.9 | 7304 ± 13.3 | 506 ± 3.02 |
| **Mn (mg/kg)** | <2 mm | 628 ± 0.78 | 875 ± 6.33 | 884 ± 1.08 | 915 ± 38.1 | 984 ± 22.9 | 1199 ± 77.7 | 57 ± 2.85 |
|  | <250 µm | 624 ± 1.65 | 863 ± 8.98 | 896 ± 5.99 | 953 ± 4.65 | 962 ± 2.25 | 1202 ± 15.1 | 63 ± 1.88 |
| **Ni (mg/kg)** | <2 mm | 329 ± 1.16 | 355 ± 1.30 | 347 ± 13.3 | 306 ± 2.03 | 287 ± 2.20 | 293 ± 2.58 | 9.23 ± 0.33 |
|  | <250 µm | 328 ± 1.76 | 357 ± 0.79 | 350 ± 9.76 | 326 ± 2.22 | 286 ± 0.64 | 329 ± 0.61 | 13.4 ± 0.26 |
| **Pb (mg/kg)** | <2 mm | 1418 ± 8.31 | 1742 ± 9.67 | 2047 ± 5.65 | 9023 ± 178 | 1714 ± 8.71 | 2922 ± 52.2 | 892 ± 23.0 |
|  | <250 µm | 1405 ± 5.30 | 1735 ± 9.41 | 2018 ± 39.7 | 10304 ± 36.5 | 1737 ± 35.1 | 3549 ± 74.9 | 1103 ± 4.95 |
| **Sb (mg/kg)** | <2 mm | < LOD | 776 ± 21.8 | 1,284 ± 11.8 | 31.9 ± 4.52 | 5983 ± 164 | 524 ± 20.4 | < LOD |
|  | <250 µm | < LOD | 747 ± 60.9 | 1,164 ± 18.9 | 51.6 ± 2.20 | 6073 ± 195 | 565 ± 30.4 | < LOD |
| **Sr (mg/kg)** | <2 mm | 90.4 ± 0.41 | 83.2 ± 0.06 | 78.8 ± 0.18 | 35.0 ± 1.30 | 46.2 ± 1.37 | 73.9 ± 2.60 | 13.3 ± 0.55 |
|  | <250 µm | 88.5 ± 0.82 | 82.2 ± 0.44 | 78.3 ± 0.96 | 39.7 ± 0.78 | 45.6 ± 0.04 | 77.4 ± 1.20 | 15.7 ± 0.08 |
| **Ti (mg/kg)** | <2 mm | 528 ± 10.3 | 512 ± 1.13 | 453 ± 1.82 | 573 ± 9.67 | 313 ± 8.61 | 406 ± 26.6 | 170 ± 7.20 |
|  | <250 µm | 566 ± 0.18 | 466 ± 28.8 | 431 ± 1.86 | 668 ± 10.2 | 308 ± 6.64 | 427 ± 5.48 | 198 ± 0.23 |
| **V (mg/kg)** | <2 mm | 40.9 ± 0.44 | 50.8 ± 0.10 | 49.6 ± 0.32 | 44.3 ± 3.20 | 47.2 ± 1.50 | 45.2 ± 2.55 | 31.6 ± 0.20 |
|  | <250 µm | 42.7 ± 0.19 | 47.4 ± 1.23 | 46.7 ± 0.68 | 47.2 ± 0.17 | 48.3 ± 2.07 | 47.2 ± 0.50 | 32.7 ± 0.20 |
| **Zn (mg/kg)** | <2 mm | 667 ± 5.51 | 1,103 ± 7.13 | 1,268 ± 12.4 | 6,555 ± 65.5 | 1,213 ± 9.18 | 1,696 ± 11.2 | 418 ± 5.30 |
|  | <250 µm | 648 ± 6.86 | 1,089 ± 19.3 | 1,290 ± 3.26 | 7,962 ± 132 | 1,199 ± 1.66 | 2,250 ± 22.8 | 408 ± 0.32 |

**Table S3:** Average ± SEM pseudo-total elemental concentrations (n = 2) in the < 2 mm, < 250 µm, < 53 µm and < 20 µm particle size fractions of grey sands from Victorian goldfields (Australia). Values below the limit of detection is indicated as “<LOD“.

| **Element** | **Particle size** | **G1** | **G2** | **G3** | **G4** | **G5** | **G6** | **G7** | **G8** | **G9** | **G10** | **G11** |
| --- | --- | --- | --- | --- | --- | --- | --- | --- | --- | --- | --- | --- |
| **Al (mg/kg)** | <2 mm | 27,419 ± 3145 | 25,686 ± 411 | 21,396 ± 1616 | 9,786 ± 516 | 26,761 ± 5591 | 11,349 ± 256 | 17,015 ± 6.74 | 22,567 ± 580 | 19,394 ± 287 | 18,535 ± 2408 | 11,760 ± 91.2 |
|  | <250 µm | 21,756 ± 347 | 21,696 ± 967 | 20,899 ± 475 | 2,5711 ± 225 | 16,186 ± 1313 | 12,415 ± 675 | 18,258 ± 781 | 24,964 ± 582 | 21,872 ± 745 | 24,549 ± 6583 | 13,763 ± 8.05 |
| **As (mg/kg)** | <2 mm | 2,542 ± 6.57 | 2,791 ± 12.0 | 1,653 ± 11.9 | 681 ± 34.5 | 2,316 ± 12.7 | 1,038 ± 2.65 | 2,924 ± 57.6 | 1,817 ± 33.4 | 1,420 ± 1.71 | 2,067 ± 34.1 | 5,089 ± 652 |
|  | <250 µm | 2,580 ± 113 | 2,705 ± 32.0 | 1,758 ± 11.4 | 1,279 ± 0.26 | 2,657 ± 16.9 | 1,053 ± 3.00 | 4,249 ± 52.6 | 1,929 ± 80.9 | 2,515 ± 17.1 | 3,353 ± 4.35 | 5,242 ± 9.33 |
| **Ba (mg/kg)** | <2 mm | 175 ± 30.3 | 126 ± 23.15 | 168 ± 17.3 | 89 ± 10.7 | 149 ± 39.8 | 70 ± 2.26 | 48 ± 4.31 | 151 ± 51.2 | 92 ± 35.1 | 109 ± 23.6 | 69 ± 1.09 |
|  | <250 µm | 122 ± 3.24 | 161 ± 15.2 | 161 ± 4.77 | 234 ± 1.13 | 124 ± 36.6 | 78 ± 6.17 | 67 ± 3.58 | 113 ± 5.26 | 120 ± 38.8 | 141 ± 38.0 | 62 ± 0.51 |
| **Ca (mg/kg)** | <2 mm | 5,241 ± 10.0 | 8,941 ± 251 | 6,157 ± 70.3 | 8,641 ± 3.00 | 6,075 ± 218 | 4,213 ± 46.3 | 7,167 ± 58.1 | 6,712 ± 18.4 | 6,145 ± 365 | 6,447 ± 41.3 | 12,416 ± 36.7 |
|  | <250 µm | 5,054 ± 13.0 | 7,335 ± 26.0 | 6,301 ± 66.0 | 9,960 ± 147 | 6,676 ± 415 | 4,478 ± 14.4 | 8,357 ± 32.5 | 6,703 ± 475 | 7,105 ± 171 | 6,883 ± 12.1 | 13,000 ± 86.1 |
| **Cd (mg/kg)** | <2 mm | < LOD | 2.23 ± 0.02 | < LOD | < LOD | 2.09 ± 0.02 | < LOD | 1.89 ± 0.03 | 2.41 ± 0.08 | 1.66 ± 0.08 | 1.83 ± 0.04 | 2.19 ± 0.03 |
|  | <250 µm | < LOD | 0.88 ± 0.01 | < LOD | < LOD | 1.08 ± 0.19 | < LOD | 0.88 ± 0.03 | 1.06 ± 0.17 | 1.02 ± 0.01 | 1.01 ± 0.01 | 0.41 ± 0.03 |
| **Co (mg/kg)** | <2 mm | 15.6 ± 1.82 | 12.9 ± 1.43 | 9.52 ± 0.07 | 9.07 ± 0.16 | 13.6 ± 0.08 | 9.54 ± 0.19 | 11.0 ± 0.21 | 14.9 ± 2.12 | 9.50 ± 0.07 | 9.60 ± 0.78 | 9.28 ± 0.36 |
|  | <250 µm | 9.09 ± 0.14 | 12.2 ± 0.01 | 10.3 ± 0.14 | 12.7 ± 0.07 | 13.3 ± 1.10 | 9.67 ± 0.05 | 15.9 ± 0.20 | 13.0 ± 1.19 | 14.9 ± 0.25 | 12.2 ± 0.20 | 9.74 ± 0.10 |
| **Cr (mg/kg)** | <2 mm | 36.6 ± 3.61 | 26.6 ± 2.42 | 37.9 ± 2.15 | 20.1 ± 1.29 | 22.7 ± 3.18 | 18.1 ± 0.06 | 12.7 ± 0.19 | 30.1 ± 4.97 | 17.1 ± 3.09 | 17.2 ± 2.06 | 32.5 ± 0.03 |
|  | <250 µm | 30.4 ± 0.39 | 37.6 ± 1.68 | 37.8 ± 0.79 | < LOD | 30.3 ± 0.72 | 18.8 ± 0.74 | 19.4 ± 0.03 | 29.2 ± 3.34 | 27.2 ± 4.63 | 27.2 ± 4.52 | 40.4 ± 0.14 |
| **Fe (mg/kg)** | <2 mm | 32,253 ± 373 | 33,526 ± 255 | 27,205 ± 229 | 18,553 ± 481 | 31,302 ± 78 | 23,838 ± 644 | 26,500 ± 126 | 33,018 ± 338 | 23,638 ± 398 | 24,682 ± 306 | 33,867 ± 468 |
|  | <250 µm | 31,707 ± 56 | 34,351 ± 235 | 28,512 ± 99 | 24,371 ± 138 | 33,716 ± 1 | 23,404 ± 141 | 34,105 ± 107 | 33,487 ± 406 | 31,768 ± 520 | 30,412 ± 285 | 34,635 ± 237 |
| **Mg (mg/kg)** | <2 mm | 11,630 ± 311 | 10,935 ± 231 | 14,280 ± 168 | 7,451 ± 200 | 8,735 ± 197 | 5,288 ± 31.8 | 7,246 ± 24.1 | 10,723 ± 275 | 7,443 ± 228 | 6,831 ± 170 | 4,912 ± 12.5 |
|  | <250 µm | 11,068 ± 27.4 | 13,064 ± 135 | 14,258 ± 459 | 10,048 ± 160 | 11,569 ± 1009 | 5,775 ± 67.1 | 9,868 ± 130 | 11,225 ± 1079 | 10,450 ± 82.8 | 8,113 ± 327 | 5,429 ± 68.3 |
| **Mn (mg/kg)** | <2 mm | 373 ± 8.39 | 373 ± 9.58 | 267 ± 5.37 | 307 ± 5.34 | 366 ± 2.42 | 336 ± 5.75 | 339 ± 0.10 | 396 ± 25.8 | 331 ± 8.15 | 333 ± 13.5 | 570 ± 7.76 |
|  | <250 µm | 309 ± 1.36 | 394 ± 0.17 | 289 ± 0.75 | 369 ± 2.27 | 397 ± 16.4 | 349 ± 0.58 | 461 ± 7.49 | 398 ± 21.3 | 471 ± 9.89 | 428 ± 13.2 | 601 ± 2.61 |
| **Ni (mg/kg)** | <2 mm | 37.0 ± 0.68 | 40.2 ± 0.01 | 40.5 ± 0.26 | 29.2 ± 0.81 | 30.1 ± 0.15 | 22.2 ± 0.18 | 29.6 ± 0.16 | 50.1 ± 2.49 | 25.2 ± 1.20 | 24.8 ± 0.78 | 14.3 ± 0.11 |
|  | <250 µm | 32.8 ± 0.41 | 38.6 ± 0.02 | 42.5 ± 0.08 | 39.6 ± 0.10 | 41.1 ± 10.5 | 22.4 ± 0.12 | 40.3 ± 0.01 | 40.7 ± 10.1 | 36.6 ± 0.22 | 30.7 ± 0.02 | 14.9 ± 0.03 |
| **Pb (mg/kg)** | <2 mm | 125 ± 11.2 | 45.2 ± 0.04 | 104 ± 4.37 | 49.7 ± 1.96 | 39.0 ± 1.77 | 70.6 ± 1.36 | 33.2 ± 1.36 | 41.2 ± 0.78 | 28.8 ± 1.54 | 45.4 ± 0.56 | 44.2 ± 0.04 |
|  | <250 µm | 106 ± 2.07 | 42.2 ± 1.16 | 106 ± 1.68 | 117 ± 0.41 | 37.0 ± 2.00 | 74.0 ± 1.82 | 50.6 ± 0.02 | 36.8 ± 0.35 | 42.3 ± 2.67 | 64.5 ± 2.55 | 46.6 ± 0.80 |
| **Sb (mg/kg)** | <2 mm | < LOD | < LOD | < LOD | < LOD | < LOD | < LOD | 5.39 ± 0.08 | < LOD | 2.64 ± 2.64 | < LOD | < LOD |
|  | <250 µm | < LOD | < LOD | < LOD | < LOD | < LOD | < LOD | 6.53 ± 1.57 | < LOD | 2.52 ± 0.48 | 3.47 ± 0.76 | < LOD |
| **Sr (mg/kg)** | <2 mm | 92 ± 0.25 | 112 ± 0.14 | 145 ± 0.48 | 165 ± 6.12 | 91.9 ± 2.32 | 57.5 ± 0.49 | 96.6 ± 4.21 | 90.2 ± 1.04 | 85.3 ± 3.47 | 89.6 ± 3.53 | 43.8 ± 0.80 |
|  | <250 µm | 90.2 ± 0.26 | 130 ± 0.63 | 154 ± 1.17 | 208 ± 1.78 | 109 ± 0.26 | 61.6 ± 0.08 | 126 ± 0.84 | 107 ± 2.45 | 116 ± 2.80 | 104 ± 2.25 | 44.8 ± 0.45 |
| **Ti (mg/kg)** | <2 mm | 218 ± 29.9 | 192 ± 18.0 | 231 ± 21.2 | 201 ± 23.9 | 98 ± 31.6 | 90 ± 2.19 | 125 ± 3.25 | 229 ± 35.0 | 186 ± 46.4 | 195 ± 25.7 | 775 ± 18.5 |
|  | <250 µm | 167 ± 1.67 | 224 ± 14.4 | 215 ± 6.54 | 314 ± 5.37 | 134 ± 38.5 | 97 ± 5.96 | 119 ± 0.83 | 129 ± 63.2 | 190 ± 42.9 | 181 ± 37.2 | 672 ± 21.9 |
| **V (mg/kg)** | <2 mm | 26.8 ± 3.43 | 24.7 ± 3.14 | 32.7 ± 3.29 | 20.8 ± 0.89 | 20.3 ± 3.80 | 13.9 ± 0.09 | 13.5 ± 0.72 | 28.4 ± 4.79 | 14.7 ± 4.83 | 15.1 ± 4.16 | 28.4 ± 1.06 |
|  | <250 µm | 22.7 ± 0.57 | 29.9 ± 2.27 | 31.6 ± 0.82 | 20.8 ± 0.89 | 23.7 ± 0.75 | 14.9 ± 0.98 | 14.8 ± 0.09 | 22.5 ± 2.69 | 23.4 ± 5.74 | 26.2 ± 5.70 | 34.3 ± 0.02 |
| **Zn (mg/kg)** | <2 mm | 90 ± 0.63 | 93 ± 0.38 | 123 ± 0.64 | 109 ± 2.90 | 94 ± 0.33 | 128 ± 1.18 | 140 ± 0.20 | 113 ± 1.12 | 88 ± 1.69 | 131 ± 1.07 | 87 ± 0.43 |
|  | <250 µm | 88 ± 0.26 | 94 ± 0.24 | 143 ± 4.49 | 156 ± 0.05 | 108 ± 8.34 | 128 ± 0.97 | 198 ± 3.66 | 106 ± 7.98 | 134 ± 3.18 | 176 ± 1.21 | 96 ± 1.70 |

**Table S4:** Average ± SEM pseudo-total elemental concentrations (n = 2) in the < 2 mm, < 250 µm, < 53 µm and < 20 µm particle size fractions of tailings from Victorian goldfields (Australia). Values below the limit of detection is indicated as “<LOD“.

| **Element** | **Particle size** | **T1** | **T2** | **T3** | **T4** | **T5** | **T6** | **T7** | **T8** | **T9** | **T10** | **T11** | **T12** |
| --- | --- | --- | --- | --- | --- | --- | --- | --- | --- | --- | --- | --- | --- |
| **Al (mg/kg)** | <2 mm | 27,322 ± 885 | 35,901 ± 1937 | 26,361 ± 740 | 19,466 ± 1072 | 41,644 ± 1114 | 38,696 ± 1,349 | 11,081 ± 1696 | 41,321 ± 1531 | 10,929 ± 45.6 | 12,013 ± 2.43 | 19,347 ± 2,174 | 15,749 ± 6,983 |
|  | <250 µm | 29,434 ± 947 | 34,390 ± 347 | 25,457 ± 4357 | 15,175 ± 2136 | 42,602 ± 6466 | 35,720 ± 1,539 | 10,338 ± 575 | 44,661 ± 2089 | 10,847 ± 325 | 15,356 ± 35.9 | 19,776 ± 1,280 | 7,354 ± 184 |
| **As (mg/kg)** | <2 mm | 843 ± 16.5 | 801 ± 1.88 | 470 ± 6.67 | 2,530 ± 8.45 | 593 ± 6.50 | 96 ± 2.86 | 840 ± 27.5 | 1,566 ± 37.2 | 5,481 ± 74.3 | 4,791 ± 16.6 | 538 ± 1.34 | 145 ± 21.0 |
|  | <250 µm | 967 ± 11.4 | 942 ± 12.7 | 592 ± 16.0 | 3,313 ± 36.5 | 581 ± 5.27 | 68 ± 2.31 | 973 ± 14.1 | 2,443 ± 12.7 | 6,418 ± 252 | 5,872 ± 123 | 569 ± 3.15 | 125 ± 0.93 |
| **Ba (mg/kg)** | <2 mm | 240 ± 1.74 | 253 ± 11.6 | 209 ± 4.08 | 662 ± 28.2 | 272 ± 20.4 | 196 ± 10.4 | 104 ± 0.93 | 46.9 ± 1.28 | 87.3 ± 3.26 | 74.1 ± 2.29 | 114 ± 14.1 | 118 ± 51.2 |
|  | <250 µm | 211 ± 2.26 | 192 ± 4.59 | 161 ± 31.4 | 718 ± 2.02 | 261 ± 55.7 | 185 ± 8.11 | 92.3 ± 6.43 | 50.6 ± 0.90 | 75.0 ± 1.60 | 70.2 ± 0.88 | 116 ± 7.15 | 56.7 ± 0.94 |
| **Ca (mg/kg)** | <2 mm | 46,929 ± 6952 | 21,424 ± 361 | 51,528 ± 766 | 157,254 ± 6212 | 11,311 ± 1059 | 2,330 ± 73.1 | 5,162 ± 70.5 | 2,703 ± 88.4 | 13,810 ± 51.8 | 13,962 ± 218 | 706 ± 0.56 | 187 ± 10.3 |
|  | <250 µm | 51,002 ± 482 | 19,642 ± 159 | 54,678 ± 278 | 175,155 ± 3715 | 10,369 ± 19.9 | 2,487 ± 24.2 | 6,157 ± 39.0 | 2,827 ± 20.0 | 14,578 ± 124 | 13,459 ± 32.0 | 698 ± 3.58 | 153 ± 8.30 |
| **Cd (mg/kg)** | <2 mm | < LOD | < LOD | < LOD | < LOD | < LOD | < LOD | < LOD | 4.28 ± 0.29 | < LOD | < LOD | < LOD | < LOD |
|  | <250 µm | < LOD | < LOD | < LOD | 0.85 ± 0.05 | < LOD | < LOD | < LOD | 1.17 ± 0.05 | < LOD | < LOD | < LOD | < LOD |
| **Co (mg/kg)** | <2 mm | 5.65 ± 0.02 | 7.05 ± 0.12 | 6.22 ± 0.10 | 16.0 ± 0.10 | 8.19 ± 0.06 | 8.19 ± 0.01 | 16.3 ± 0.99 | 13.1 ± 0.08 | 10.8 ± 0.08 | 10.1 ± 0.27 | 21.2 ± 0.22 | 8.93 ± 0.44 |
|  | <250 µm | 5.48 ± 0.05 | 6.93 ± 0.05 | 5.41 ± 0.12 | 16.4 ± 0.07 | 7.90 ± 0.12 | 7.71 ± 0.05 | 19.2 ± 2.23 | 13.3 ± 0.13 | 11.7 ± 0.10 | 11.2 ± 0.03 | 21.9 ± 0.14 | 8.58 ± 0.36 |
| **Cr (mg/kg)** | <2 mm | 21.1 ± 0.04 | 27.5 ± 1.34 | 19.7 ± 0.68 | 17.5 ± 0.76 | 33.9 ± 1.23 | 35.7 ± 0.07 | 13.2 ± 2.62 | 263 ± 5.03 | 43.7 ± 1.87 | 47.0 ± 3.03 | 28.4 ± 1.67 | 21.0 ± 4.78 |
|  | <250 µm | 26.1 ± 0.49 | 31.6 ± 0.43 | 21.8 ± 3.31 | 16.7 ± 2.12 | 42.1 ± 5.49 | 38.5 ± 0.96 | 11.8 ± 0.50 | 146 ± 0.76 | 46.5 ± 1.20 | 50.3 ± 1.20 | 28.9 ± 0.89 | 15.8 ± 0.27 |
| **Fe (mg/kg)** | <2 mm | 16,780 ± 500 | 18,369 ± 323 | 14,537 ± 85.1 | 29,935 ± 721 | 25,725 ± 252 | 34,762 ± 4,008 | 41,212 ± 2,351 | 68,886 ± 3587 | 38,852 ± 1,364 | 39,487 ± 3065 | 31,848 ± 138 | 17,713 ± 591 |
|  | <250 µm | 17,416 ± 24.4 | 19,746 ± 84.3 | 14,957 ± 164 | 29,537 ± 492 | 24,593 ± 495 | 25,842 ± 218 | 45,573 ± 39.9 | 47,763 ± 379 | 38,614 ± 461 | 37,597 ± 11.2 | 33,390 ± 390 | 17,282 ± 28.1 |
| **Mg (mg/kg)** | <2 mm | 41,941 ± 6521 | 43,292 ± 89.2 | 55,692 ± 1588 | 22,118 ± 77.1 | 18,896 ± 1478 | 5,949 ± 133 | 1,161 ± 66.5 | 2,300 ± 42.4 | 6,061 ± 17.5 | 5,427 ± 51.4 | 2,974 ± 164 | 1,016 ± 352 |
|  | <250 µm | 53,534 ± 498 | 49,220 ± 337 | 70,274 ± 214 | 13,827 ± 5.4 | 18,910 ± 253 | 6,818 ± 15.4 | 1,360 ± 34.3 | 3,109 ± 36.2 | 6,830 ± 6.37 | 6,254 ± 44.5 | 2,847 ± 39.1 | 586 ± 10.8 |
| **Mn (mg/kg)** | <2 mm | 382 ± 1.95 | 203 ± 0.88 | 341 ± 0.29 | 4,730 ± 160 | 228 ± 5.95 | 110 ± 1.07 | 630 ± 321 | 380 ± 5.24 | 601 ± 8.58 | 569 ± 11.1 | 157 ± 1.02 | 66 ± 9.64 |
|  | <250 µm | 406 ± 2.82 | 216 ± 0.64 | 394 ± 0.68 | 6,302 ± 179 | 220 ± 2.82 | 117 ± 1.61 | 416 ± 23.6 | 411 ± 3.07 | 640 ± 8.88 | 613 ± 0.25 | 157 ± 1.52 | 58 ± 1.86 |
| **Ni (mg/kg)** | <2 mm | 9.0 ± 0.15 | 10.6 ± 0.54 | 10.8 ± 0.03 | 42.5 ± 0.34 | 17.2 ± 0.09 | 13.4 ± 0.20 | 23.3 ± 2.52 | 35.7 ± 1.28 | 16.7 ± 0.24 | 17.2 ± 0.76 | 41.6 ± 0.82 | 14.7 ± 0.86 |
|  | <250 µm | 9.4 ± 0.11 | 11.0 ± 0.26 | 10.4 ± 0.48 | 46.9 ± 0.54 | 17.2 ± 0.66 | 12.4 ± 0.42 | 25.5 ± 0.62 | 38.1 ± 0.98 | 17.9 ± 0.08 | 18.1 ± 0.19 | 42.2 ± 0.51 | 13.2 ± 0.17 |
| **Pb (mg/kg)** | <2 mm | < LOD | < LOD | < LOD | 145 ± 1.29 | 13.2 ± 0.05 | 18.7 ± 1.54 | 78.5 ± 0.82 | 32.6 ± 2.04 | 55.5 ± 0.53 | 47.5 ± 1.51 | 174 ± 5.52 | 84.0 ± 20.8 |
|  | <250 µm | < LOD | < LOD | < LOD | 160 ± 0.52 | < LOD | 7.29 ± 0.55 | 85.1 ± 1.83 | 29.3 ± 0.73 | 56.8 ± 0.31 | 48.4 ± 0.58 | 188 ± 1.07 | 56.6 ± 0.27 |
| **Sb (mg/kg)** | <2 mm | < LOD | < LOD | < LOD | 7.31 ± 0.30 | < LOD | < LOD | < LOD | < LOD | < LOD | < LOD | 34.7 ± 5.25 | < LOD |
|  | <250 µm | < LOD | < LOD | < LOD | 4.91 ± 1.02 | < LOD | < LOD | < LOD | < LOD | < LOD | < LOD | 34.5 ± 0.57 | < LOD |
| **Sr (mg/kg)** | <2 mm | 2,707 ± 4.48 | 1,834 ± 76.6 | 1,736 ± 22.0 | 1,840 ± 54.2 | 630 ± 21.8 | 84.2 ± 1.04 | 39.2 ± 1.20 | 35.5 ± 6.76 | 49.1 ± 0.67 | 68.5 ± 24.5 | 15.9 ± 0.33 | 4.6 ± 4.62 |
|  | <250 µm | 2,642 ± 13.9 | 1,451 ± 4.19 | 1,871 ± 6.75 | 2,278 ± 57.0 | 503 ± 1.20 | 86.1 ± 0.44 | 45.6 ± 0.23 | 22.0 ± 0.50 | 49.6 ± 1.96 | 46.2 ± 0.85 | 16.3 ± 0.21 | < LOD |
| **Ti (mg/kg)** | <2 mm | 299 ± 13.9 | 436 ± 41.4 | 331 ± 8.68 | 333 ± 31.0 | 425 ± 19.6 | 479 ± 26.1 | 56.0 ± 17.2 | 822 ± 15.8 | 700 ± 44.7 | 659 ± 48.9 | 154 ± 15.8 | < LOD |
|  | <250 µm | 348 ± 18.2 | 405 ± 32.0 | 291 ± 56.4 | 326 ± 25.0 | 449 ± 79.6 | 523 ± 21.4 | 37.0 ± 2.60 | 729 ± 3.35 | 780 ± 73.4 | 729 ± 22.5 | 157 ± 6.74 | 54.3 ± 0.78 |
| **V (mg/kg)** | <2 mm | 29.5 ± 0.78 | 39.4 ± 1.23 | 53.6 ± 0.89 | 21.1 ± 1.24 | 35.5 ± 1.17 | 47.1 ± 1.66 | 18.2 ±1.63 | 163 ± 6.49 | 36.8 ± 4.23 | 40.6 ± 5.12 | 23.9 ± 1.89 | 19.0 ± 5.81 |
|  | <250 µm | 39.8 ± 0.99 | 48.0 ± 1.15 | 34.0 ± 4.60 | 23.7 ± 1.97 | 54.2 ± 6.54 | 53.7 ± 1.68 | 16.8 ± 0.63 | 94.6 ± 0.15 | 40.7 ± 0.24 | 41.4 ± 0.60 | 24.6 ± 0.91 | 12.5 ± 0.21 |
| **Zn (mg/kg)** | <2 mm | < LOD | < LOD | < LOD | 122 ± 15.1 | < LOD | < LOD | 195 ± 1.55 | 61.4 ± 0.86 | 89.6 ± 0.72 | 84.9 ± 0.30 | 164 ± 3.38 | 56.8 ± 2.06 |
|  | <250 µm | < LOD | < LOD | < LOD | 137 ± 5.57 | < LOD | < LOD | 202 ± 0.76 | 75.2 ± 0.24 | 101 ± 0.33 | 96.6 ± 0.81 | 170 ± 0.61 | 52.6 ± 2.16 |

**Table S5: As, Cd, Pb and Sb in vitro bioaccessibility (IVBA) in the <250 µm particle size fractions of mine waste from Victorian goldfields (Australia). <LOD = below level of detection.**

| **Sample type** | | **As IVBA (mg/kg)** | | **As IVBA (%)** | | **Cd IVBA (mg/kg)** | | **Cd IVBA (%)** | | **Pb IVBA (mg/kg)** | | **Pb IVBA (%)** | | **Sb IVBA (mg/kg)** | | **Sb IVBA (%)** | |
| --- | --- | --- | --- | --- | --- | --- | --- | --- | --- | --- | --- | --- | --- | --- | --- | --- | --- |
|  |  | **Mean** | **SEM** | **Mean** | **SEM** | **Mean** | **SEM** | **Mean** | **SEM** | **Mean** | **SEM** | **Mean** | **SEM** | **Mean** | **SEM** | **Mean** | **SEM** |
| **Calcine sands** | **C1** | 6,456 | 21.5 | 77.6 | 0.26 | 1.78 | 0.03 | 10.2 | 0.15 | 699 | 4.91 | 49.8 | 0.35 | <LOD | <LOD | <LOD | <LOD |
|  | **C2** | 6,842 | 82.4 | 45.7 | 0.55 | 2.25 | 0.02 | 12.0 | 0.09 | 1,216 | 7.82 | 70.1 | 0.45 | 28.7 | 0.15 | 3.85 | 0.02 |
|  | **C3** | 5,850 | 15.0 | 45.4 | 0.12 | 2.42 | 0.00 | 12.8 | 0.01 | 1,479 | 5.8 | 73.3 | 0.29 | 23.1 | 0.19 | 1.98 | 0.02 |
|  | **C4** | 5,152 | 76.6 | 43.4 | 0.65 | 7.23 | 0.11 | 14.0 | 0.22 | 7,823 | 14.3 | 75.9 | 0.14 | 1.39 | 0.00 | 2.69 | 0.00 |
|  | **C5** | 3,878 | 37.6 | 44.2 | 0.43 | 1.93 | 0.03 | 10.7 | 0.16 | 1,377 | 4.57 | 79.3 | 0.26 | 49.2 | 0.97 | 0.81 | 0.02 |
|  | **C6** | 5,211 | 49.5 | 46.0 | 0.44 | 4.44 | 0.06 | 18.7 | 0.24 | 3,012 | 59.5 | 84.9 | 1.68 | 17.9 | 0.20 | 3.16 | 0.04 |
|  | **C7** | 492 | 0.67 | 13.5 | 0.02 | 1.13 | 0.00 | 3.91 | 0.01 | 51.2 | 0.25 | 4.64 | 0.02 | <LOD | <LOD | <LOD | <LOD |
| **Grey sands** | **G1** | 1,845 | 9.50 | 71.5 | 0.37 | <LOD | <LOD | <LOD | <LOD | 26.5 | 0.29 | 25.0 | 0.27 | <LOD | <LOD | <LOD | <LOD |
|  | **G2** | 1,679 | 10.2 | 62.1 | 0.38 | 0.17 | 0.02 | 19.1 | 1.80 | 26.9 | 0.37 | 63.9 | 0.87 | <LOD | <LOD | <LOD | <LOD |
|  | **G3** | 1,043 | 17.3 | 59.3 | 0.98 | <LOD | <LOD | <LOD | <LOD | 25.7 | 0.75 | 24.3 | 0.71 | <LOD | <LOD | <LOD | <LOD |
|  | **G4** | 461 | 6.97 | 36.1 | 0.54 | <LOD | <LOD | <LOD | <LOD | 22.2 | 1.09 | 19.0 | 0.93 | <LOD | <LOD | <LOD | <LOD |
|  | **G5** | 1,253 | 32.4 | 47.2 | 1.22 | 0.12 | 0.01 | 10.9 | 0.94 | 18.9 | 0.19 | 51.0 | 0.51 | <LOD | <LOD | <LOD | <LOD |
|  | **G6** | 479 | 0.05 | 45.5 | 0.01 | <LOD | <LOD | <LOD | <LOD | 13.7 | 0.16 | 18.5 | 0.21 | <LOD | <LOD | <LOD | <LOD |
|  | **G7** | 1,819 | 16.0 | 42.8 | 0.38 | 0.23 | 0.00 | 26.1 | 0.24 | 27.7 | 1.30 | 54.7 | 2.56 | 0.97 | 0.01 | 14.8 | 0.15 |
|  | **G8** | 812 | 1.02 | 42.1 | 0.05 | 0.26 | 0.00 | 24.1 | 0.45 | 22.1 | 0.12 | 60.0 | 0.32 | <LOD | <LOD | <LOD | <LOD |
|  | **G9** | 1,073 | 2.55 | 42.7 | 0.10 | 0.21 | 0.00 | 20.3 | 0.30 | 25.5 | 0.24 | 60.2 | 0.56 | 0.83 | 0.01 | 33.10 | 0.32 |
|  | **G10** | 1,196 | 10.9 | 35.7 | 0.33 | 0.46 | 0.20 | 45.7 | 19.73 | 46.7 | 14.2 | 72.4 | 21.98 | 0.72 | 0.21 | 20.73 | 5.91 |
|  | **G11** | 786 | 2.70 | 15.0 | 0.05 | 0.09 | 0.00 | 22.5 | 1.16 | 26.1 | 0.44 | 56.0 | 0.94 | <LOD | <LOD | <LOD | <LOD |
| **Tailings** | **T1** | 627 | 2.37 | 64.9 | 0.25 | 0.03 | 0.00 | 7.31 | 0.74 | <LOD | <LOD | <LOD | <LOD | <LOD | <LOD | <LOD | <LOD |
|  | **T2** | 610 | 11.0 | 64.7 | 1.16 | <LOD | <LOD | <LOD | <LOD | <LOD | <LOD | <LOD | <LOD | <LOD | <LOD | <LOD | <LOD |
|  | **T3** | 358 | 24.4 | 60.4 | 4.12 | <LOD | <LOD | <LOD | <LOD | <LOD | <LOD | <LOD | <LOD | <LOD | <LOD | <LOD | <LOD |
|  | **T4** | 1,305 | 21.8 | 39.4 | 0.66 | 0.16 | 0.01 | 18.2 | 1.50 | 79.1 | 4.77 | 49.4 | 2.97 | 0.43 | 0.04 | 8.75 | 0.78 |
|  | **T5** | 193 | 4.51 | 33.3 | 0.78 | <LOD | <LOD | <LOD | <LOD | <LOD | <LOD | <LOD | <LOD | <LOD | <LOD | <LOD | <LOD |
|  | **T6** | 14.7 | 0.44 | 21.5 | 0.64 | <LOD | <LOD | <LOD | <LOD | 4.41 | 0.03 | 60.4 | 0.46 | <LOD | <LOD | <LOD | <LOD |
|  | **T7** | 160 | 0.68 | 16.4 | 0.07 | 0.11 | 0.01 | 3.16 | 0.36 | 11.3 | 3.30 | 13.2 | 3.88 | <LOD | <LOD | <LOD | <LOD |
|  | **T8** | 371 | 10.3 | 15.2 | 0.42 | 0.06 | 0.00 | 5.23 | 0.15 | 13.7 | 1.30 | 46.9 | 4.43 | <LOD | <LOD | <LOD | <LOD |
|  | **T9** | 918 | 2.82 | 14.3 | 0.04 | 0.09 | 0.00 | 36.7 | 0.05 | 34.2 | 0.21 | 60.2 | 0.37 | <LOD | <LOD | <LOD | <LOD |
|  | **T10** | 1,077 | 247 | 18.3 | 4.20 | <LOD | <LOD | <LOD | <LOD | 36.6 | 6.44 | 75.5 | 13.3 | <LOD | <LOD | <LOD | <LOD |
|  | **T11** | 66.1 | 1.11 | 11.6 | 0.20 | <LOD | <LOD | <LOD | <LOD | 58.6 | 0.05 | 31.1 | 0.03 | 0.49 | 0.02 | 1.43 | 0.06 |
|  | **T12** | 7.57 | 0.26 | 6.07 | 0.21 | <LOD | <LOD | <LOD | <LOD | 10.4 | 0.06 | 18.5 | 0.11 | <LOD | <LOD | <LOD | <LOD |

**Relationship between elemental concentrations in the < 2 mm and < 250 µm particle fractions**

The relationship between As, Cd, Pb and Sb concentrations in the <2 mm and <250 µm particle fractions was further explored to assess enrichment of these elements in the incidentally ingestible fraction (Fig. S1). The HIL A values for As, Pb and Cd are indicated as the red dotted line, while the adopted Sb HIL A value is indicated with orange dotted line. Dotted black lines in Fig. S1 represents the 1:1 relationship for visual comparison. According to Fig. S1A, As elemental enrichment in the <250 µm particle fractions was 0.86 - 1.77. Variability and range of As elemental enrichment between the two particle size fractions observed in this study are similar with results in Ollson et al., [9]. Fig. S1B-1D showed a similar pattern of variable elemental enrichment for Cd (0.19 - 1.17), Pb (0.67 - 2.35) and Sb (0.67 - 1.21). Overall, enrichment in the smaller particle size for these four elements was small for majority of the samples, which agrees with the findings of Ollson et al., [9].


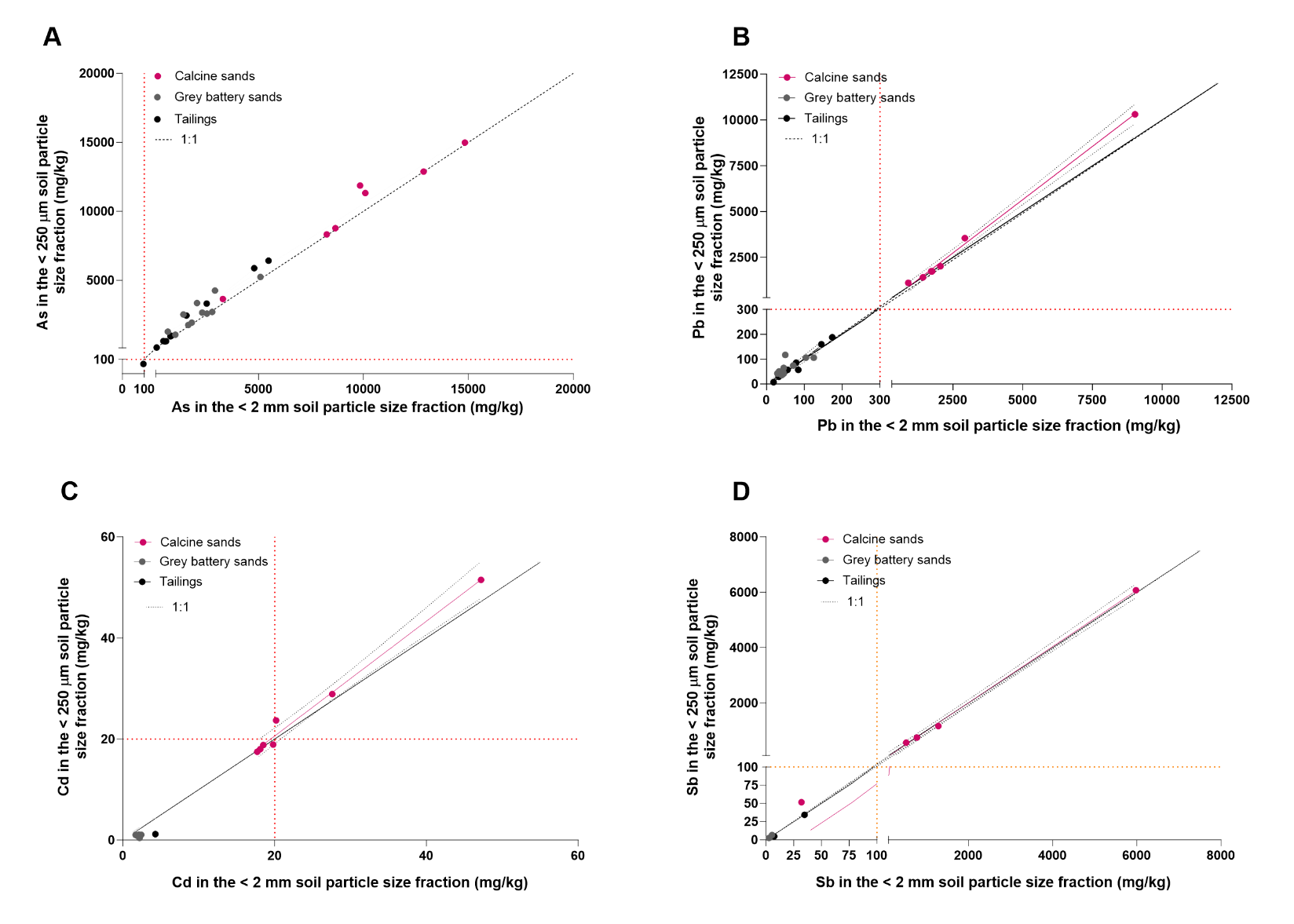


**Fig. S1:** Relationship between elemental concentrations in the < 2 mm and < 250 µm particle fractions in legacy gold mining waste from the Victorian goldfields (Australia): As (Fig S1A), Pb (Fig. S1B), Cd (Fig. S1C), and Sb (Fig. S1D). The red dotted lines represent the Health based Investigation Level A (HIL A) according to the National Environmental Protection Measure for the Assessment of Site Contamination (NEPM-ASC, 2013): As: 100 mg/kg, Pb: 300 mg/kg, Cd: 20 mg/kg. The orange dotted line is a value of 100 mg/kg was adopted for Sb because of its structural similarity with As because soil guideline value for this element is currently unavailable.


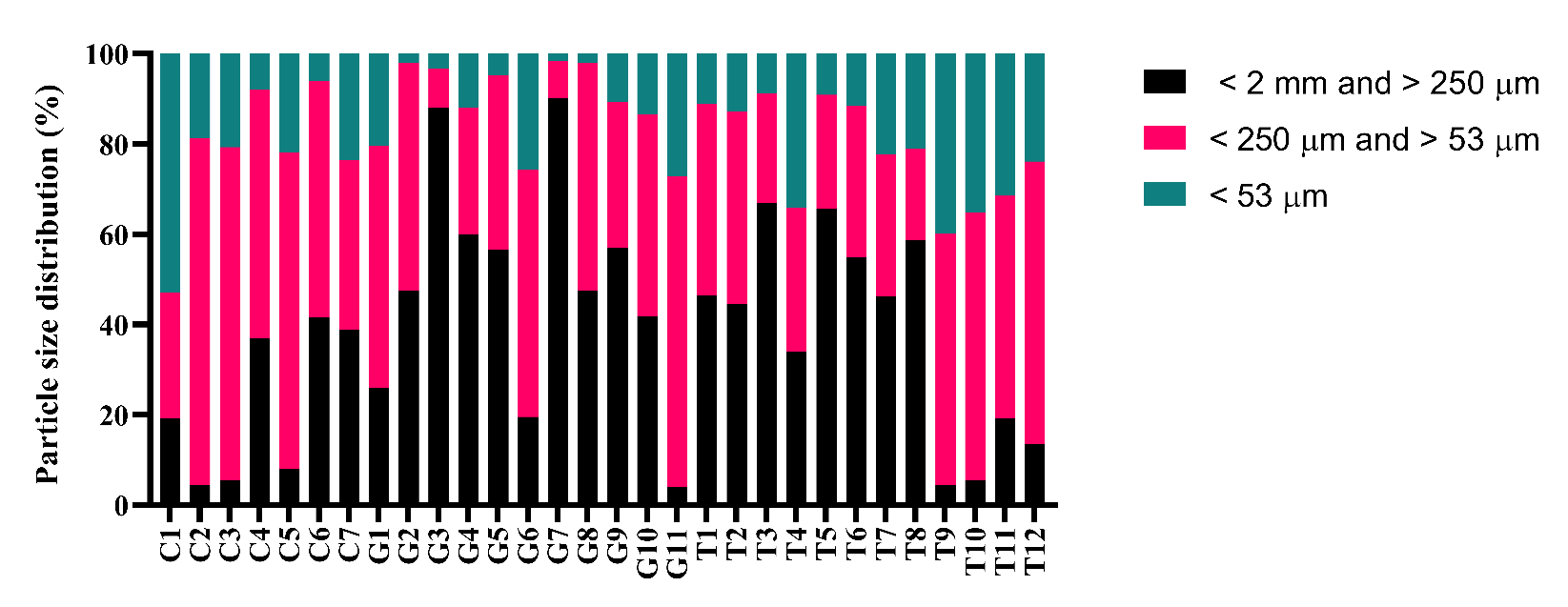


**Fig. S2:** Particle size distribution in calcine sands (C1-C7), grey sands (G1-G11) and tailings (T1-T12) from the Victorian goldfields, Australia**.**

**Relationship between total and bioaccessible As concentration**

The relationship between total and bioaccessible As concentration (mg/kg and %) was analyzed using linear regression in Fig S2. Fig. S2 shows that when all samples were considered together, a strong and significant relationship was observed between total and bioaccessible As concentrations (r2 = 0.85, slope: 0.48, p <0.001). However, when the three mining wastes were investigated separately, strong, and significant (p <0.05) relationships were observed between total and bioaccessible As for calcine sands (r2 = 0.70, slope: 0.49) and tailings (r2 = 0.62, slope: 0.16) only. In contrast, high variability resulted in a poor relationship between total and bioaccessible As for grey battery sands (r2 = 0.17, slope: 0.16, p = 0.21). The strength of these relationships show that total metal(loid) concentration may not be a suitable predictor of bioaccessibility.

**Fig. S3:** Relationship between total and bioaccessible As concentration (mg/kg)

**Fig. S4:** Relationship between total (mg/kg) and bioaccessible As (%) in calcine sands, grey battery sands and tailings from the Victorian goldfields, Australia.

**Methods for As (K-edge), Sb (K-edge) and Pb (L_3_-edge) X-ray Absorption Spectroscopy (XAS) data collection and analysis**

**Sample processing**

XAS was performed on the <250 µm particle fraction of samples at the Materials Research Collaborative Access Team 10-BM for As and Sb (Kropf et al., 2010), and 10-ID for Pb (Segre et al., 2000), Advanced Photon Source (Argonne National Laboratory). The storage ring operated at 7 GeV and in top-up mode and on both 10-BM and 10-ID, a liquid N_2_-cooled double crystal Si(111) monochromator was tuned to select incident photon energies and a platinum-coated mirror was used for harmonic rejection. Samples were prepared for XAS by grinding in agate mortar and pestle, mixed with polyvinylpyrrolidone (PVP) as binder, hand-pressed into a 1-cm pellet, and mounted on Kapton tape for measurement.

**As XAS analysis and LCF**

Arsenic XAS data collection at 10-BM was measured at the As K edge (11,867 eV) using a 4-element Vortex fluorescence detector with several layers of aluminum foil shield to suppress fluorescence from other elements (i.e., iron) in the samples. For each unknown sample, three to five scans were collected in both transmission and fluorescence mode with a sodium arsenate pellet as a reference sample. Energy was calibrated by shifting spectra eV to match first derivative inflection point of sodium arsenate to be 11,874 eV (Beak et al., 2009). Data were then background subtracted and converted to k space for EXAFS region analysis. Data processed for EXAFS analysis were k^3^-weighted and all e0 set to 11,874 eV for uniform k range start energy.

The spectra were analyzed by combining principal component analysis (PCA), target transform testing (TT) and linear combination fits (LCF) with As species standard spectra (Manceau et al., 2002). Spectra were fit in 1^st^ derivative XANES LCF for As oxidation state. LCF of derivative XANES was performed at -20 to 30 eV from e0, constraints of all weights between 0 and 1 and force weights to sum to 1. Fitting with single standards for each oxidation state, arsenopyrite for As(-I), As(III) ads ferrihydrite (AsIII), and As(V) ads ferrihydrite (AsV).

PCA in combination with TT provided statistical well-ness of fit for selecting the relevant spectra to be used in LCF analysis of the EXAFS spectra. PCA and TT analyses were conducted using SIXPack (Webb, 2005) and LCF analysis by Athena (Ravel and Newville, 2005). PCA on As standards in EXAFS fitting range 3-10 k included 25 total standards. Of these, 15 components required by minimum IND value but visual inspection of component spectra had 12 components with any signal above noise (Stable #). LCF of EXAFS spectra were performed on k^3^ weighted EXAFS from k range 3-10 k (Å^-1^), constraints of all weights between 0 and 1 and force weights to sum to 1. LCF of the EXAFS data for species identification and relative abundance (Foster and Kim, 2014). Few samples were unable to be fit, calcine sands C1-C6 and spectra are plotted in Figure S4 without LCF models. These samples contained artefacts in data and/or unknown As species as a major phase which precluded accurate models so none are reported. The suspected artefact can be seen as a signal peak in C1-C6 at 10 k.

Abbreviations used are ‘ads’ for ‘adsorbed’ and ‘coppt’ for ‘coprecipitated’. Standard library of As standards were utilized in LCF of 25 candidate As standards (Stevens et al. 2018): As(V) coprecipitated calcite (Alexandratos et al. 2007), As(III) ads Al_2_O_3_, As(III) ads ferrihydrite, As(III) ads_montmorillonite, As(V) ads birnessite, As(V) ads ferrihydrite, As(V) ads gibbsite, As(V) ads goethite, arseniosiderite, As(III) coppt Pyrite, arsenolite (As_2_O_3_), arsenopyrite, loellingite (FeAs_2_), orpiment, parascorodite, Pb-arsenate (PbHAsO_4_), pharmacosiderite (KFeAsO_4_OH), realgar, scorodite (source 1), scorodite, source 2 (FeAsO_4_), beudantite (1Pb: 1As), K-jarosite coppt Pb and As, ferric arsenate, elemental As(0), and Na-Jarosite coppt As (Paktunc and Dutrizac 2003), hörnesite (Mg_3_(AsO_4_)_2_ x 8 H_2_O) (Voigt and Brantley 1997).

**As LCF**

**Table S6:** As XANES 1^st^ deriv. XANES LCF for oxidation state. Standard error ±5%

| **Name** | **As (-I) species** | **As(III)** | **As(V)** | **sum** | **R factor** |
| --- | --- | --- | --- | --- | --- |
| **C1** | 0.0% | 0.0% | 100.0% | 100% | 0.0522761 |
| **C2** | 0.0% | 0.0% | 100.0% | 100% | 0.0236265 |
| **C3** | 0.0% | 0.0% | 100.0% | 100% | 0.0253616 |
| **C4** | 0.0% | 0.0% | 100.0% | 100% | 0.0540054 |
| **C5** | 0.0% | 1.5% | 98.5% | 100% | 0.028972 |
| **C6** | 0.0% | 0.0% | 100.0% | 100% | 0.0550155 |
| **C7** | 0.0% | 0.0% | 100.0% | 100% | 0.031571 |
| **G1** | 0.0% | 0.0% | 100.0% | 100% | 0.0234397 |
| **G2** | 0.0% | 0.0% | 100.0% | 100% | 0.0435582 |
| **G3** | 0.0% | 0.0% | 100.0% | 100% | 0.0358595 |
| **G4** | 18.2% | 0.0% | 81.8% | 100% | 0.0259442 |
| **G5** | 9.2% | 0.0% | 90.8% | 100% | 0.028333 |
| **G6** | 9.6% | 0.0% | 90.4% | 100% | 0.0241767 |
| **G7** | 15.4% | 0.0% | 84.6% | 100% | 0.0189164 |
| **G8** | 0.0% | 0.0% | 100.0% | 100% | 0.0321271 |
| **G9** | 0.0% | 0.0% | 100.0% | 100% | 0.0238619 |
| **G10** | 11.2% | 0.0% | 88.8% | 100% | 0.0264434 |
| **G11** | 68.3% | 0.0% | 31.7% | 100% | 0.0255949 |
| **T1** | 0.0% | 4.8% | 95.2% | 100% | 0.0204562 |
| **T2** | 0.0% | 5.9% | 94.1% | 100% | 0.022341 |
| **T3** | 0.0% | 10.6% | 89.4% | 100% | 0.0249007 |
| **T4** | 9.1% | 0.0% | 90.9% | 100% | 0.0247582 |
| **T5** | 0.0% | 2.5% | 97.5% | 100% | 0.0226561 |
| **T6** | 0.0% | 0.0% | 100.0% | 100% | 0.0276723 |
| **T7** | 0.0% | 0.0% | 100.0% | 100% | 0.0211631 |
| **T8** | 61.4% | 0.0% | 38.6% | 100% | 0.0332727 |
| **T9** | 69.0% | 0.0% | 31.0% | 100% | 0.0203739 |
| **T10** | 71.5% | 0.0% | 28.5% | 100% | 0.017735 |
| **T11** | 0.0% | 0.0% | 100.0% | 100% | 0.0611795 |
| **T12** | 0.0% | 0.0% | 100.0% | 100% | 0.0580844 |

**
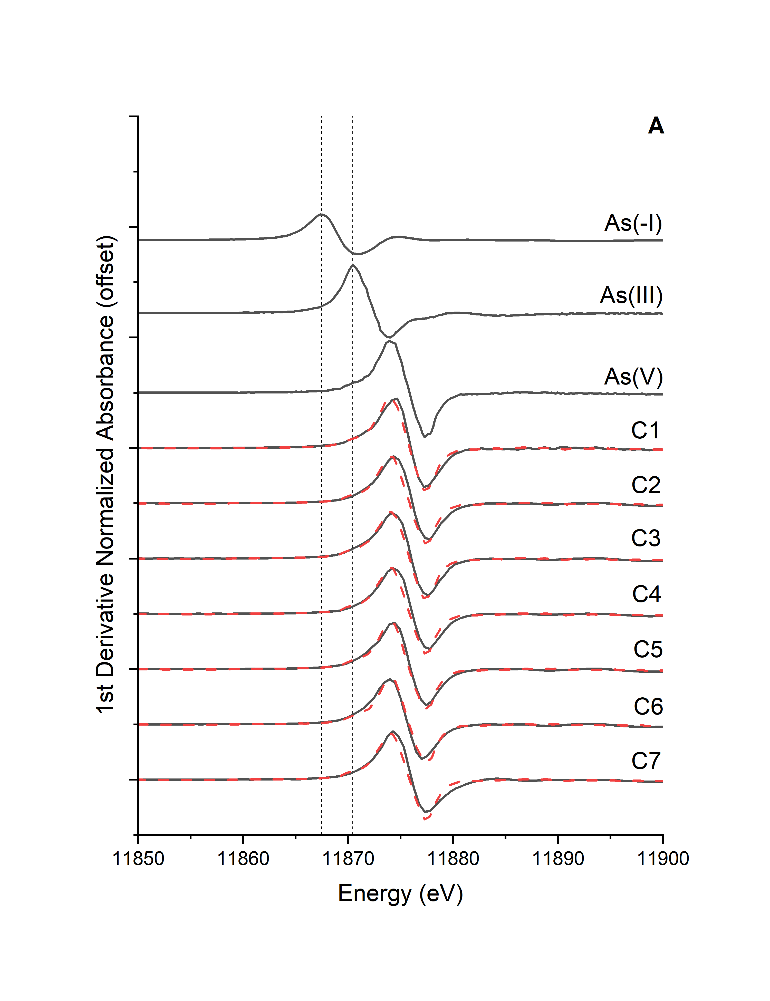

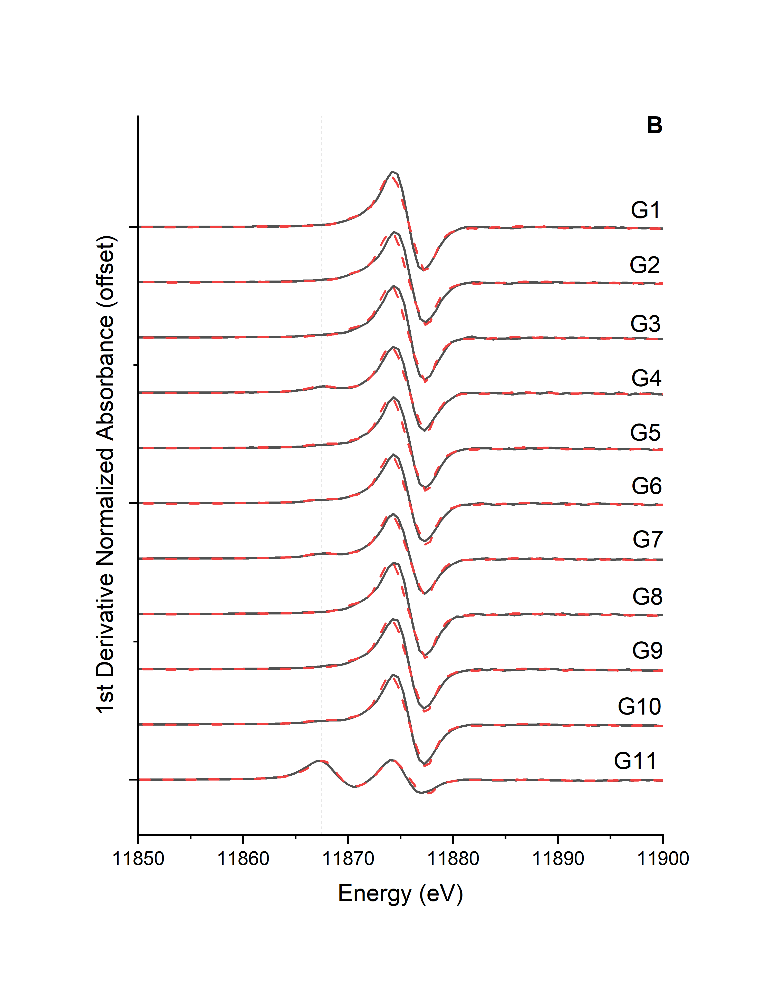

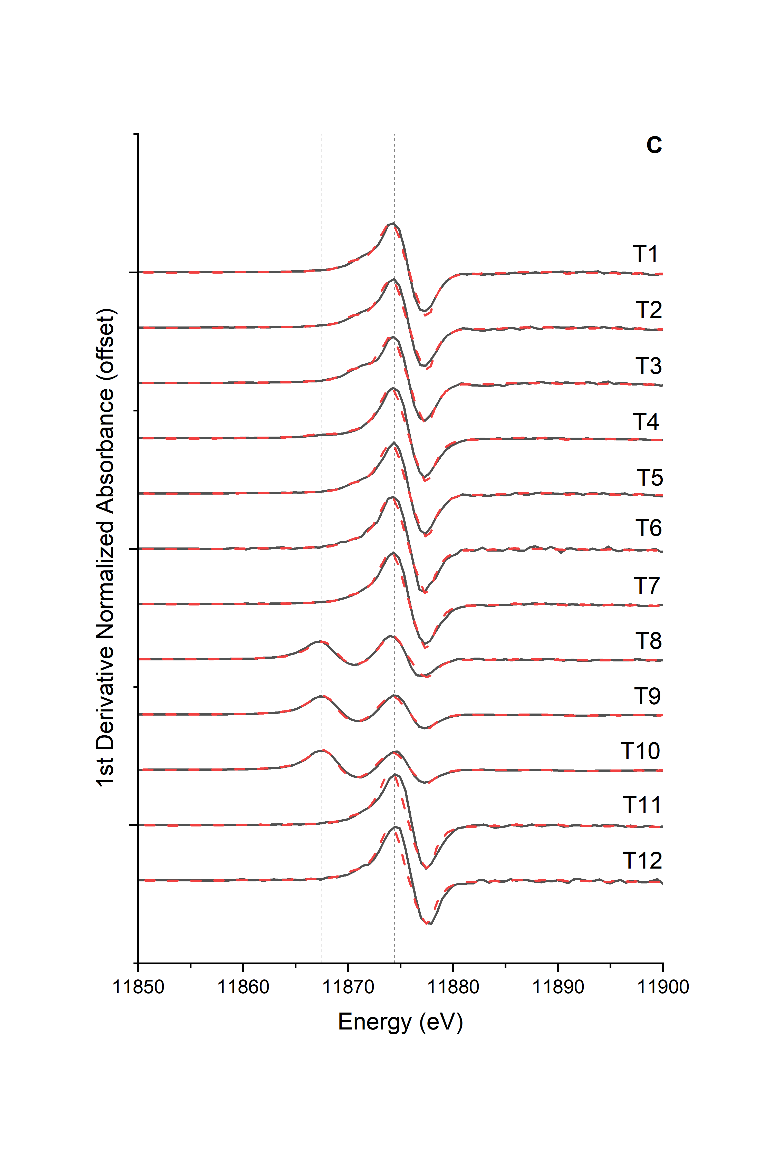
**

**Fig. S5:** As XANES LCF in 1^st^ derivative for oxidation state modelling only. Data in solid black line, fit in dashed red line. Relative abundances of As oxidation modelled using single standard for each oxidation state, arsenopyrite for As(-I), As(III) ads ferrihydrite for As(III), and As(V) ads ferrihydrite for As(V).

**Table S7:** Arsenic (As) XAS standard spectra (n = 25) selected for EXAFS LCF (in bold) of Victorian goldfields samples. Both PCA spoil values (R values) and visual comparison were used to identify standards to use with EXAFS LCF (shown in bold)

| As standard | R value |
| --- | --- |
| **arseniosiderite** | **0.04946** |
| arsenolite (As_2_O_3_) | 0.47849 |
| **arsenopyrite** | **0.08015** |
| **As(III) ads Al_2_O_3_** | **0.51268** |
| **As(III) ads Ferrihydrite** | **0.65401** |
| As(III) ads_montmorillonite | 0.58154 |
| As(III) coppt Pyrite | 0.82875 |
| As(V) ads birnessite | 0.10043 |
| **As(V) ads ferrihydrite** | **0.01417** |
| **As(V) ads gibbsite** | **0.07091** |
| **As(V) ads goethite** | **0.1931*** |
| **As(V) coppt calcite** | **0.04759** |
| As(V) coppt Na-jarosite | 0.03947 |
| elemental As(0) | 0.67458 |
| K-Jarosite coppt with Pb, As | 0.07153 |
| K-Jarosite coppt with Pb, As, source 2 | 0.07811 |
| loellingite (FeAs_2_) | 0.58736 |
| orpiment | 0.71538 |
| parascorodite | 0.05482 |
| Pb-arsenate (PbHAsO4) | 0.05785 |
| pharmacosiderite (KFeAsO_4_OH) | 0.07868 |
| **realgar** | **0.60501** |
| **scorodite** (FeAsO_4_) | **0.03367** |
| scorodite (FeAsO_4_), source 2 | 0.09919 |
| yukonite (ferric arsenate) | 0.04914 |
| **hörnesite (Mg_3_(AsO_4_)_2_ x 8 H_2_O** | **0.03268** |

***high noise in standard spectrum**

**Table S8:** Linear Combination Fitting (LCF) results of As K^3^-weighted EXAFS of Victorian goldfields samples with As standard library. Standard error is ± 5%

| **Sample** | **Arsenopyrite** | **As(III) ads Aluminum oxide** | **As(V) ads gibbsite** | **As(V) ads goethite** | **As(V) ads ferrihydrite** | **scorodite** | **arseniosiderite** | **hornesite** | **Sum** | **R factor** |
| --- | --- | --- | --- | --- | --- | --- | --- | --- | --- | --- |
| **C1** |  |  |  |  | 50% |  |  | 50% | 100% | 0.10980 |
| **C2** |  |  |  |  | 59% |  |  | 41% | 100% | 0.04619 |
| **C3** |  |  |  |  | 58% |  |  | 42% | 100% | 0.03565 |
| **C4** |  |  |  |  | 50% |  |  | 50% | 100% | 0.03936 |
| **C5** |  |  |  |  | 62% |  |  | 38% | 100% | 0.04213 |
| **C6** |  |  |  |  | 60% |  |  | 40% | 100% | 0.03431 |
| **C7** |  |  |  |  | 100% |  |  |  | 100% | 0.05385 |
| **G1** |  |  |  |  | 16% | 20% | 64% |  | 100% | 0.02781 |
| **G2** |  |  |  | 12% | 88% |  |  |  | 100% | 0.02975 |
| **G3** |  |  | 70% | 30% |  |  |  |  | 100% | 0.08654 |
| **G4** | 14% |  |  |  | 52% | 34% |  |  | 100% | 0.11006 |
| **G5** | 5% |  |  |  | 58% | 38% |  |  | 100% | 0.04235 |
| **G6** | 11% |  | 17% |  | 72% |  |  |  | 100% | 0.07017 |
| **G7** | 15% |  |  |  | 60% | 25% |  |  | 100% | 0.05039 |
| **G8** |  |  | 26% |  | 74% |  |  |  | 100% | 0.06713 |
| **G9** |  |  | 8% |  | 92% |  |  |  | 100% | 0.03915 |
| **G10** | 15% |  |  |  | 85% |  |  |  | 100% | 0.06406 |
| **G11** | 67% |  |  |  | 34% |  |  |  | 100% | 0.06440 |
| **T1** |  |  | 60% | 40% |  |  |  |  | 100% | 0.08379 |
| **T2** |  |  | 60% | 40% |  |  |  |  | 100% | 0.08379 |
| **T3** |  | 20% | 64% | 17% |  |  |  |  | 100% | 0.13748 |
| **T4** | 8% |  | 50% |  | 42% |  |  |  | 100% | 0.03161 |
| **T5** |  |  | 51% |  | 49% |  |  |  | 100% | 0.07876 |
| **T6** |  |  |  | 16% | 84% |  |  |  | 100% | 0.50956 |
| **T7** |  |  |  |  | 65% | 35% |  |  | 100% | 0.04175 |
| **T8** | 60% |  |  |  | 40% |  |  |  | 100% | 0.27249 |
| **T9** | 79% |  |  | 21% |  |  |  |  | 100% | 0.12518 |
| **T10** | 77% |  | 23% |  |  |  |  |  | 100% | 0.06790 |
| **T11** |  |  |  |  | 12% | 88% |  |  | 100% | 0.04454 |
| **T12** |  |  | 79% | 21% |  |  |  |  | 100% | 0.29442 |


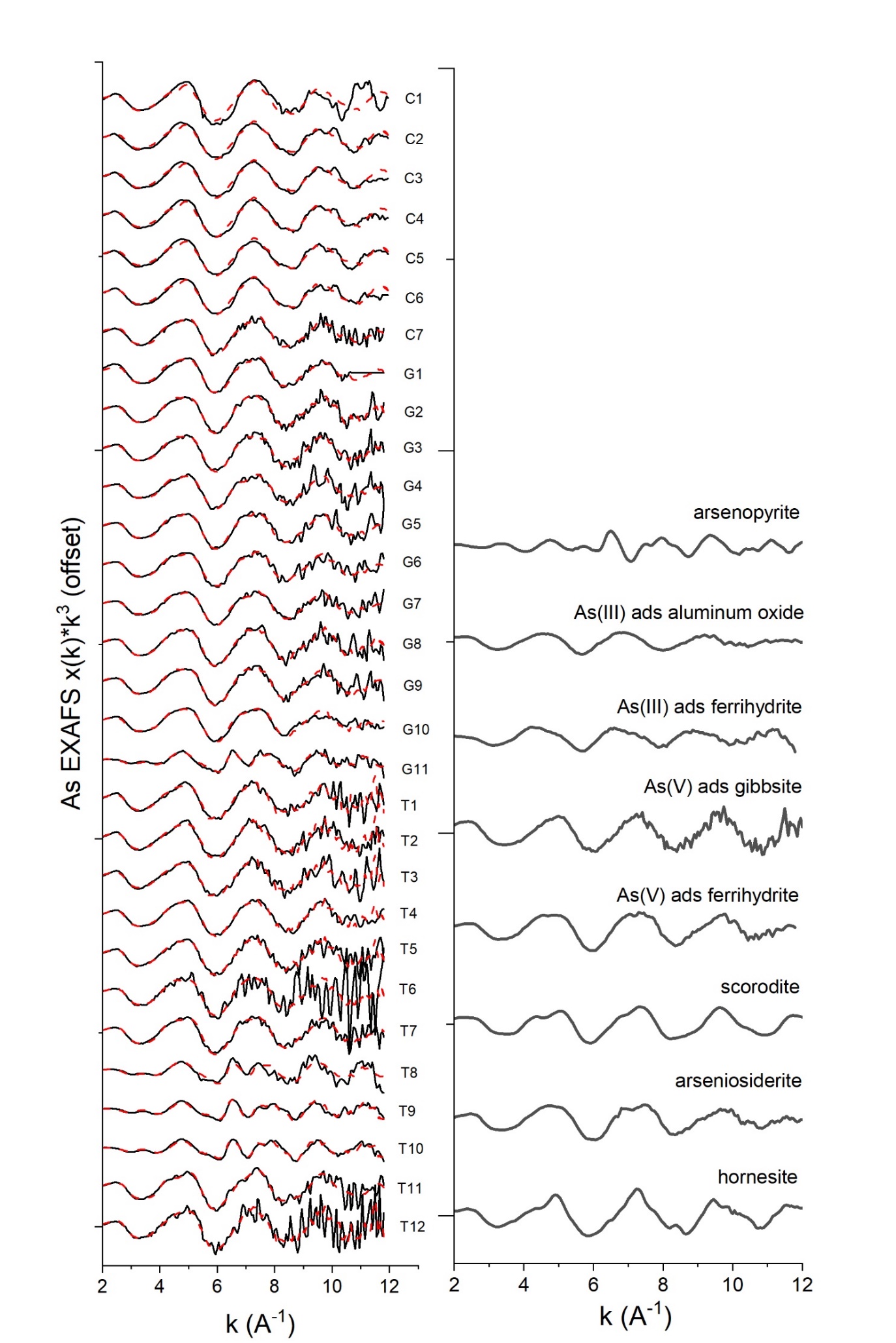


**Fig. S6:** Linear Combination Fitting (LCF) results of As K^3^-weighted EXAFS of Victorian gold mining wastes. Data in solid black line, fit in dashed red line. Model parameters are given in Tables S6 – S8.


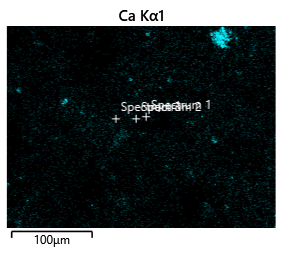

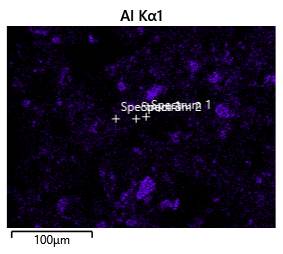

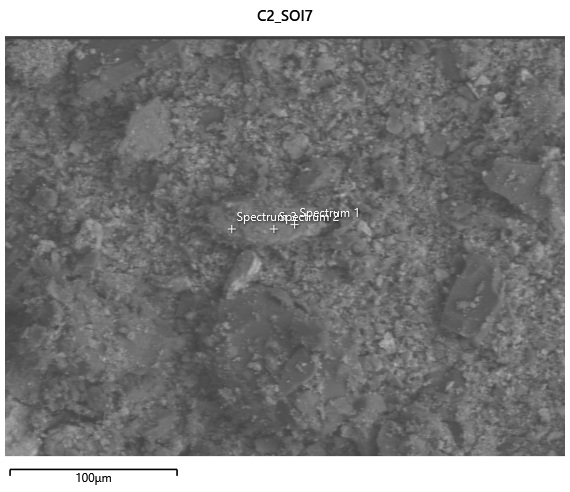

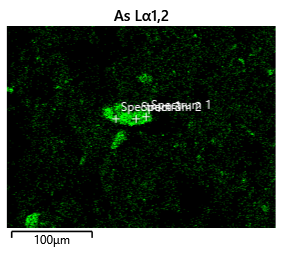

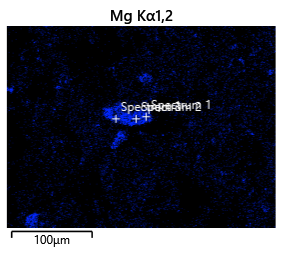

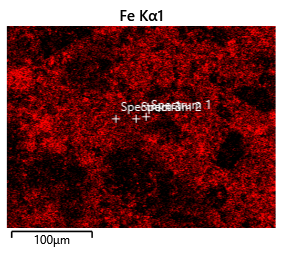

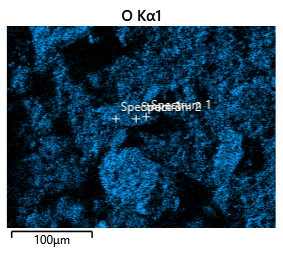


**I**

**H**

**G**

**F**

**E**

**D**

**B**

**A**

**C**


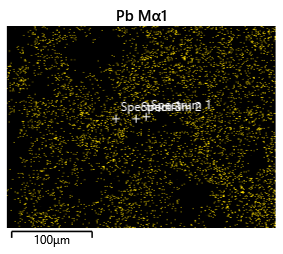

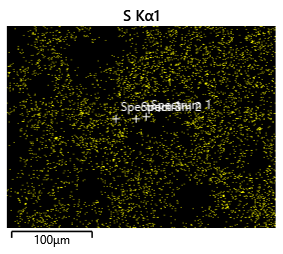


**Fig. S7.** Scanning Electron Microscopy (SEM) and spatial EDS mapping of calcine sand sample C2. Image A shows the electron image of the sample and images B-I show elemental maps. Note maps B and C show close correlation between As and Mg. Maps D-I show other elements with no correlation to As.


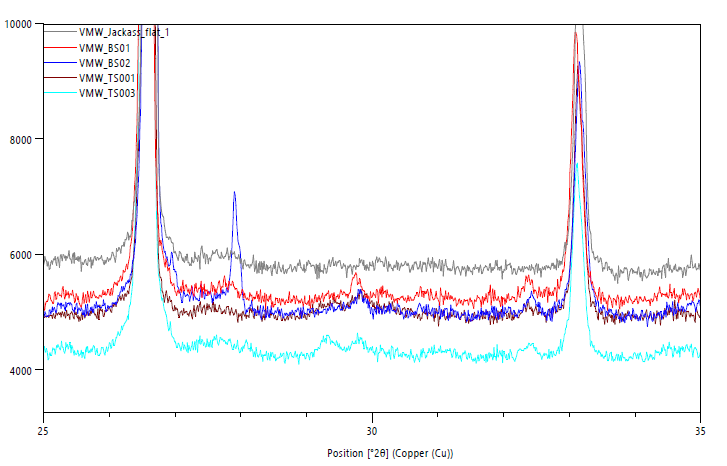


**Fig. S8:** X-ray Diffractograms (XRD) analysis of calcine sands (C1-C6) showing peaks at 32.43 and 29.82 2Ɵ as matching the two major peaks for an anhydrous Mg_3_(AsO_4_)_2_ mineral (COD # 96-210-6371). These peaks are absent from sample C1.

**
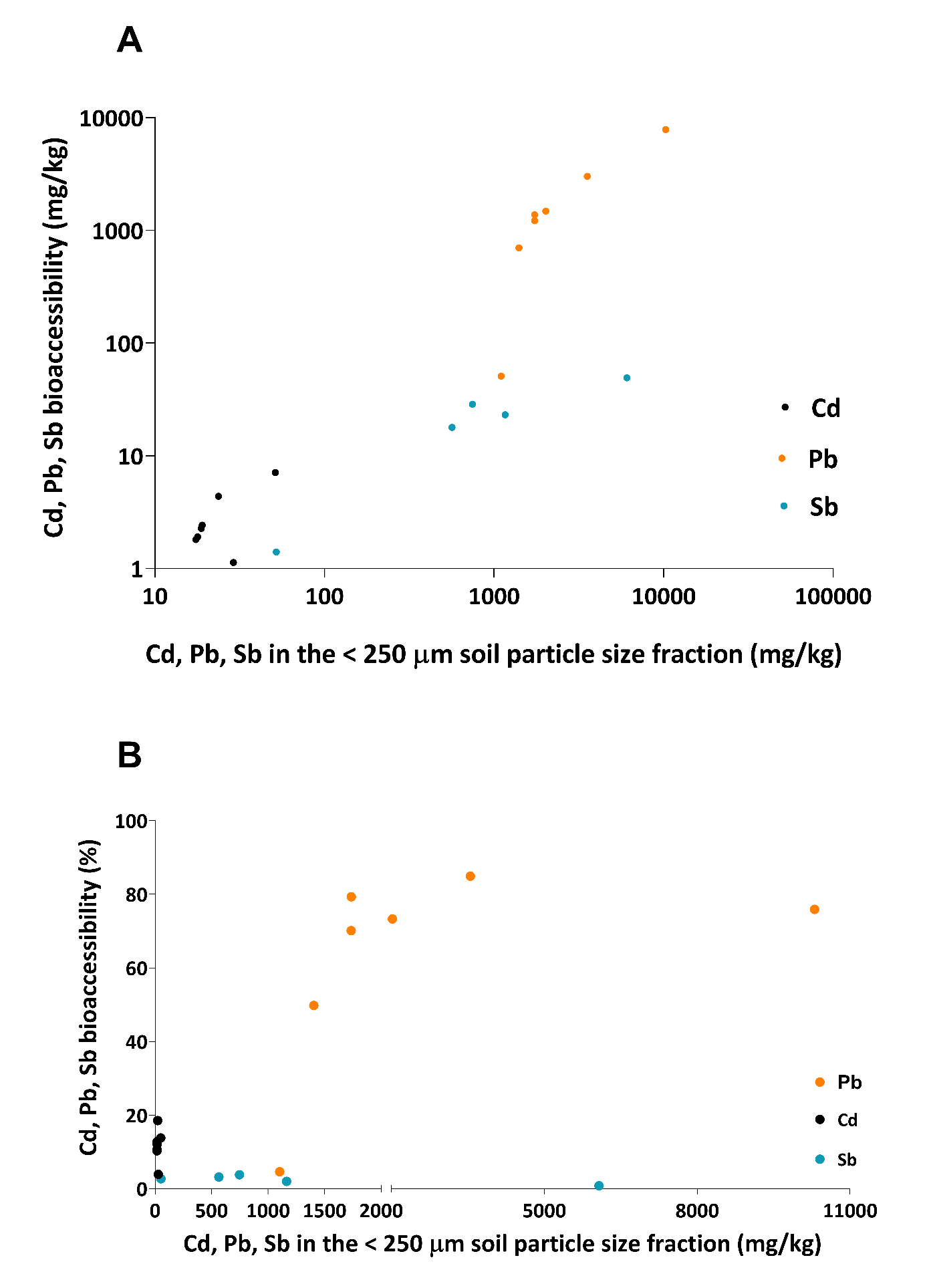
**

**Fig. S9:** Relationship between total (mg/kg) and bioaccessible Cd, Pb and Sb (A: mg/kg; B: %) in calcine sands, grey sands and tailings from the Victorian goldfields, Australia.


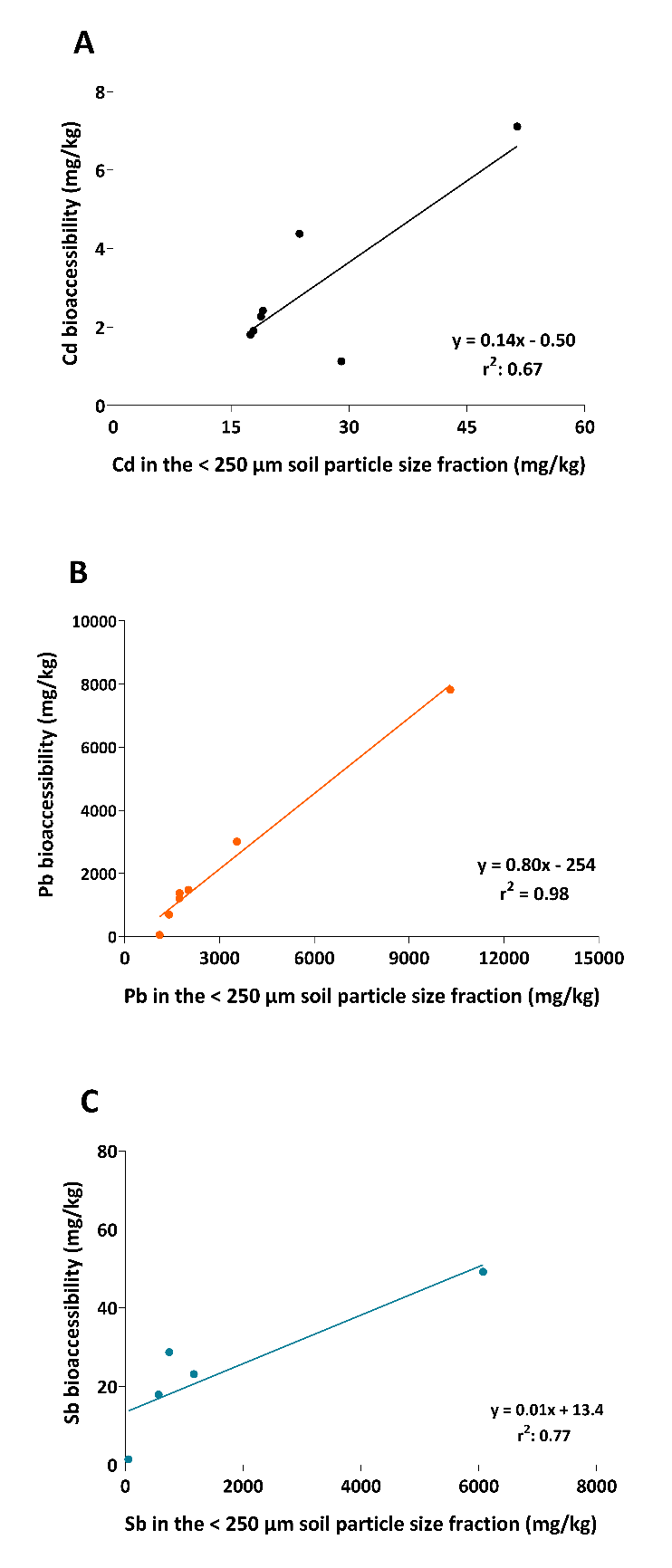


**Fig. S10:** Simple linear regression showing the relationship between total and bioaccessible co-contaminants in calcine sands (< 250 µm soil particle fraction) from legacy gold mining impacted Victorian goldfields.

**Pb XAS analysis and LCF**

Lead XAS data collection at 10-ID utilized a Si(111) mono with ring operating at 7 Ge to tune energy to the Pb L_3_-edge (13035 eV). Sample and foil were measured in transmission in kiethley ion gas chambers and fluorescence measured using a Mirion-Canberra 7-element Ge detector at 45° incident to the sample. For each sample, three to five scans were collected in both transmission and fluorescence mode with a Pb foil for reference sample. Calibration was performed by assigning the first derivative inflection point of Pb foil scan to 13035 eV (Beak et al., 2009). LCF of Pb XANES utilized the 1^st^ derivative norm(E) from -20 to 50 eV from e0, constraints of all weights between 0 and 1 and sum of weights normalized to 1.

**Table S9:** Identification of Lead (Pb) phases in Victorian gold mining Wastes. Data fit as 1^st^ derivative Pb XANES Linear Combination Fits. Estimated systematic error ± 5%.

| **Sample** | **Pb ads goethite** | **Pb ads ferrihydrite** | **Pb ads hydroxyapatite** | **Pb citrate** | **Anglesite** | **Plumbojarosite** | **PbO** | **sum** | **e0 shift** | **R factor** |
| --- | --- | --- | --- | --- | --- | --- | --- | --- | --- | --- |
| **C1** | 54% | - | - | 34% | 12% | - | - | 1 | 0.057 | 0.00388 |
| **C2** | 45% | - | - | 44% | 11% | - | - | 1 | 0.125 | 0.00493 |
| **C3** | 31% | - | - | - | 11% | 58% | - | 1 | 0.188 | 0.00874 |
| **C4** | - | - | - | 36% | 20% | 29% | 15% | 1 | 0.237 | 0.00205 |
| **C5** | 48% | - | - | 39% | - | 14% | - | 1 | 0.233 | 0.00178 |
| **C6** | - | 30% | 60% | - | 9% | - | - | 1 | 0.210 | 0.00140 |
| **C7** | 48% | - | - | 42% | 11% | - | - | 1 | 0.233 | 0.00472 |
| **T11** | 34% | - | 52% | - | 14% | - | - | 1 | -0.51 | 0.01882 |

**
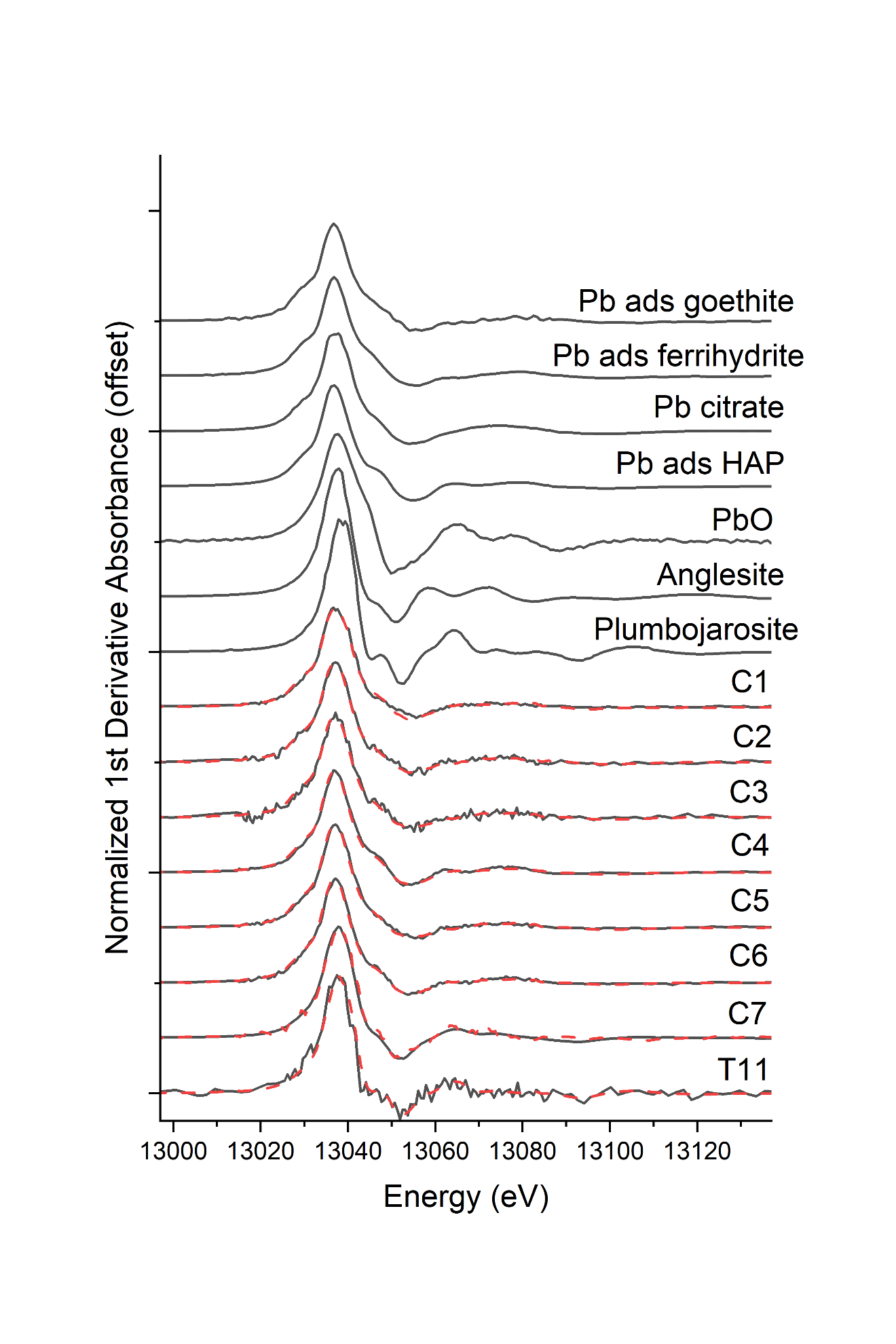
**

**Fig. S11:** Lead (Pb) L_3_-edge x-ray near edge absorption data for all standards used in final fits. Data in solid black line, fit in dashed red line. Fit statistics and relative abundances of standard contributions may be found in table #. Abbreviations in standards are Ads. for adsorbed, HAP for hydroxyapatite.

**Sb XAS analysis and LCF**

Samples were measured for Sb K-edge XAS at Sector 10-BM using a Si(111) water-cooled monochromator at Argonne National Lab Advanced Photon Source (APS). Samples were prepared by grinding in agate mortar and pestle, hand pressed into pellets in a 7-mm diameter die and held between Kapton tape. Samples were measured at room temperature in fluorescence and transmission with a vortex 4-element Be window detector or keithley ion chambers purged with N_2_ gas. All scans were energy calibrated with Sb foil transmission measurement of downbeam from sample position. Data were merged and processed utilizing Larch GUI and Athena for normalization, energy calibration and linear combination analysis with Sb standards.

Sb standards collected simultaneous to samples include: K Sb-tartrate, Sb_2_S_3_, Sb_2_O_3_, KSb(OH)_6_, and natural minerals of tetrahedrite (Cu,Fe)_12_Sb_4_S_13_, stibnite (Sb_2_S_3_). Only Sb_2_S_3_, Sb_2_O_3_, and KSb(OH)_6_ were used in LCF. Fitting range of -20 to 50 eV above the edge was used provided all standards as options. Only the standard KSb(OH)6 provided the best fits for all samples (Fig. S5). Fitting statistics of R-factor are provided in Table of LCF parameters (Table S10).


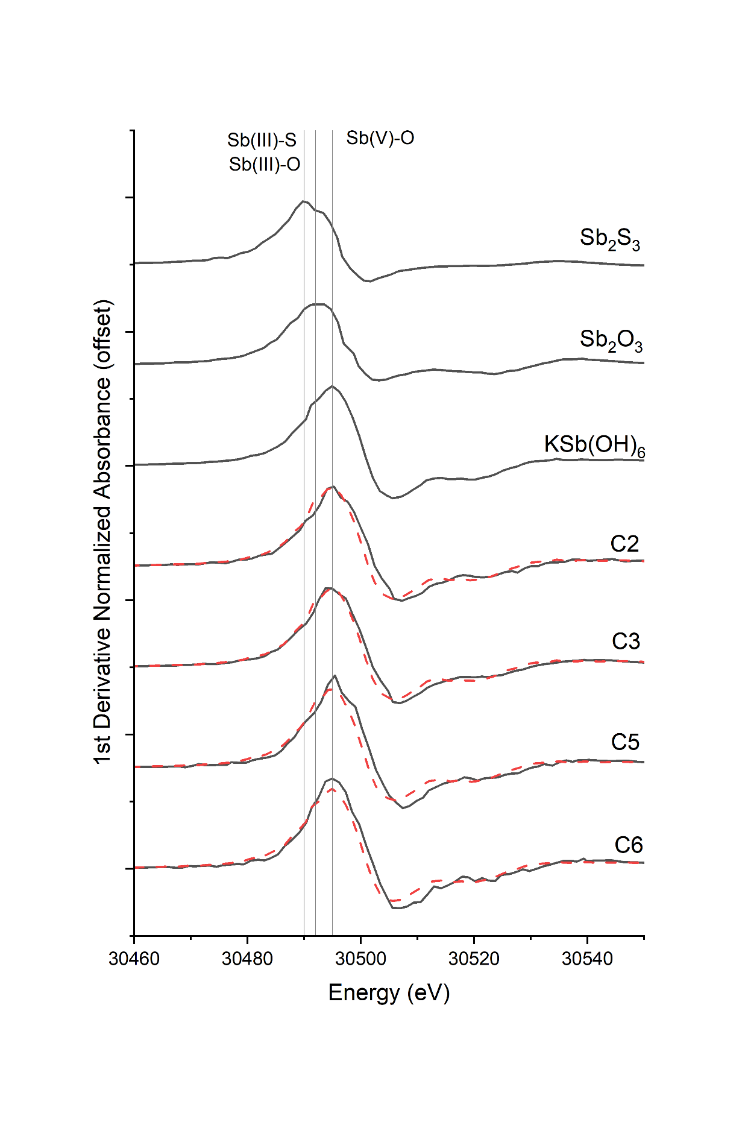


**Fig. S12:** Antimony (Sb) K-edge x-ray near edge absorption (XANES) data for all standards used in final fits. Data in solid black line, fit in dashed red line. Fit statistics and relative abundances of standard contributions may be found in Table S10.

**Table S10:** Identification of Antimony (Sb) phases in Victorian gold mining Wastes. Data fit as 1^st^ derivative Sb XANES Linear Combination Fits. Estimated error ± 5%.

| **Sample** | **Sb_2_S_3_** | **Sb_2_O_3_** | **KSb(OH)_6_** | **sum** | **e0 shift** | **R factor** |
| --- | --- | --- | --- | --- | --- | --- |
| **C2** | - | - | 100% | 1 | 0 | 0.04334 |
| **C3** | - | - | 100% | 1 | 0 | 0.05522 |
| **C5** | - | - | 100% | 1 | 0 | 0.02269 |
| **C6** | - | - | 100% | 1 | 0 | 0.04310 |
